# Exploring semiconductor potential: novel boron-based Ti_3_AlC_2_ and Ti_4_AlN_3_ MAX phase composites with tunable band gaps†

**DOI:** 10.1039/d4na00738g

**Published:** 2024-11-12

**Authors:** Md. Shahinoor Alam, Mohammad Asaduzzaman Chowdhury, Md. Saiful Islam, Md. Moynul Islam, Md. Abdus Sabur, Md. Masud Rana

**Affiliations:** a Department of Mechanical Engineering, Dhaka University of Engineering and Technology Gazipur 1707 Bangladesh majshahin4282@gmail.com; b Department of Chemistry, Bangladesh Army University of Engineering and Technology, Qadirabad Cantonment Natore-6431 Bangladesh; c Pilot Plant and Process Development Centre, Bangladesh Council of Scientific and Industrial Research (BCSIR) Dhanmondi Dhaka-1205 Bangladesh

## Abstract

This research focuses on synthesizing chemically and thermally stable novel *in situ* Ti_4_AlN_3_ and Ti_3_AlC_2_ MAX phase reinforced boron-based composites using hot pressed and inert sintering processes, enabling a sizeable and wider bandgap for semiconductor applications. The study found that the MAX phase is formed from 0.2% to 2.9% in fabricated samples with increasing sintering temperatures from 950 °C to 1325 °C. As the sintering temperature increases, the percentage of crystallinity in Ti_4_AlN_3_ MAX phase reinforced boron-based composites increases from 69.14% to 89.88%, while in Ti_3_AlC_2_ MAX phase reinforced boron-based composites, it increases from 71.02% to 77.86%. And the energy bandgap shows a declining trend from 2.33 eV to 1.78 eV for Ti_4_AlB_2_N sample composites and 2.60 eV to 2.40 for Ti_4_AlB_2_C sample composites. The UV-vis test for boron-based Ti_4_AlN_3_ and Ti_3_AlC_2_ MAX phase composites shows an absorbance rate ranging from 0.065 a.u. to 0.63 a.u. and 0.008 to 2.4 a.u. respectively with increasing sintering temperature. Tuning these bandgap variations for Ti_4_AlN_3_ and Ti_3_AlC_2_ MAX phase reinforced boron-based composites with sintering temperature allows for customization of the material's optical absorption and emission spectra, which is important for semiconductor properties and for electronic and optoelectronic devices.

## Abstract 2

Here we have synthesized Ti_4_AlN_3_ and Ti_3_AlC_2_ MAX phase reinforced boron-based composites with tunable bandgaps for electronic and optoelectronic applications.

## Graphical abstract

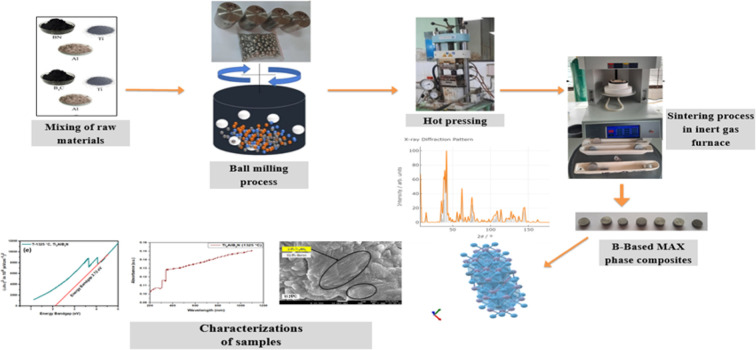

## 1. Introduction

MAX phase composites with various metals, metal oxides and metal borides such as zirconium boride (ZrB_2_), silicon boride (SiB), alumina (Al_2_O_3_), boron (B), boron carbide (B_4_C), boron nitride (BN), and titanium carbide (TiC) have been gaining significant attention in recent years due to their promising properties and semiconductor applications.^1,2^ A wider and sizeable band gap in such semiconductor materials offers several advantages in electronic and optoelectronic devices. The advantageous properties of these types of semiconductor materials include higher breakdown voltage, lower intrinsic carrier concentration, improved thermal stability, radiation resistance, suitability for optoelectronic applications, high-frequency operation, and reduced sensitivity to temperature variations.^3,4^ Recently, Avinashi *et al.* reported that 2-D novel materials and their composites are widely used in many areas such as energy storage, gas sensors, catalysis, and biomedical applications including bio-imaging, drug delivery, therapies, biosensors, tissue engineering, and antibacterial reagents.^5^ The nanocomposites of ZnO : SnO_2_ were synthesized by incorporating SnO_2_ to ZnO, where the dielectric constant was found to increase.^6^ Similarly, nano–micro composites of CdO–Al_2_O_3_ were fabricated by adding varying amounts of nanostructured CdO to microstructured Al_2_O_3_, which also led to enhanced dielectric constants.^7^ Additionally, Zankat A. *et al.* studied the frequency- and temperature-dependent electrical properties of ZnO–SnO_2_ nanocomposites, revealing that these properties are influenced by both temperature and frequency, and attributed the variations to oxygen vacancies, defects, disorder, and structural parameters.^8^

MAX phases are a fascinating class of layered ternary carbides and nitrides. These materials possess a unique combination of metallic and ceramic properties making them attractive for various applications including high-temperature ceramics, wear-resistant coatings, and parts for energy conversion systems.^9–11^ Their inherent properties like good electrical conductivity, thermal stability, and machinability allow them to be tailored for specific needs.^9^ Among the MAX phases, titanium aluminum carbide (Ti_3_AlC_2_) and titanium aluminum nitride (Ti_4_AlN_3_) stand out for their unique combination of mechanical, electrical, and thermal properties.^12,13^ However, most of the MAX phases including Ti_3_AlC_2_ and Ti_4_AlN_3_ are unstable and decompose above 1300 °C. Nayebi *et al.* utilized the Ti_3_AlC_2_ phase to lower the sintering temperature and produce TiB_2_-based composites using spark plasma sintering methods.^14^ In addition, pure Ti_3_AlC_2_ and Ti_4_AlN_3_ MAX phases exhibit brittleness, fluctuating coefficients of thermal expansion (CTE),^15^ and high chemical reactivity, which limit their applications in semiconductor devices. Incorporating boron as amorphous boron (a-B) into these MAX phases further enhances their performance, particularly in semiconductor applications where materials with tailored electrical conductivity, and thermal and chemical stability are crucial.^16^ Amorphous boron is a promising material with unique properties including high hardness, excellent wear resistance, and interesting electrical and electronic properties.^17,18^ The potential advantages of a “Ti_3_AlC_2_ and Ti_4_AlN_3_ MAX phase composite with amorphous boron” over Ti_3_AlC_2_ and Ti_4_AlN_3_ MAX phases alone in energy storage, optoelectronics, and nanoelectronic devices could be attributed to the unique properties that amorphous boron may bring to the composite material. Due to the enhanced electrical conductivity, tailored band gap, improved thermal conductivity, enhanced mechanical properties, higher specific capacity in energy storage, improved stability and reliability, compatibility with nanofabrication techniques, and versatility in design 2D materials, boron-based Ti_3_AlC_2_ and Ti_4_AlN_3_ MAX phase composites might be more advantageous in these applications.^19^

Several studies have explored the development of MAX phase composites to achieve superior properties. For instance, Wozniak *et al.* fabricated MAX phase/SiC composites demonstrating improved mechanical properties compared to individual phases.^20^ Nadeem *et al.* investigated tailoring the electronic properties of Mo_2_GaC through cationic substitution, highlighting the potential for property modification through composite formation.^21^ However, synthesizing pure, dense a-B-based MAX phase composites is challenging due to their complex structure and high melting point. Again, a significant challenge lies in developing a synthesis method to achieve a uniform dispersion of boron within the MAX phase composites. Moreover, characterizing the microstructure and properties of such composites to understand the interplay between the phases is crucial. A notable gap exists in the synthesis and characterization of *in situ* Ti_3_AlC_2_ and Ti_4_AlN_3_ MAX phase reinforced composites. Moreover, the incorporation of boron and in particular the self-generated boron into these MAX phases and its effects on microstructure and properties relevant to semiconductor applications remain unexplored. Bridging this gap is essential for advancing our understanding of MAX phase composites and unlocking their full potential in semiconductor technology. This research work seeks to address this research gap by synthesizing and characterizing Ti_3_AlC_2_ and Ti_4_AlN_3_ MAX phase reinforced boron composites, by addressing the self-generated and integrated boron and its impact on material properties, thereby contributing to the advancement of semiconductor applications in nano and optoelectronic devices. With this background, it is expected that (i) chemically and thermally stable Ti_3_AlC_2_ and Ti_4_AlN_3_ MAX phase reinforced boron composites will be produced using a hot pressed inert sintering process and (ii) semiconductor properties of tunable band gaps and high career mobility will be obtained through the synthesis of MAX phase reinforced amorphous boron composites suitable for electronic and optoelectronic devices as compared to traditional Si, Ge, CdSe, and ZnO-based semiconductor materials. This research is unique in that it is the first attempt to open up new insights for the experimental fabrication of a novel boron carbide-based MAX phase, Ti_3_AlBC, and boron-nitride-based MAX phase, Ti_3_AlBN, which has been proven theoretically by the DFT computational approach.^16,22^ Nonetheless, better knowledge is still required to synthesize boron-based carbide and nitride MAX phases. As we saw throughout the experimental investigation, boron is induced and MAX phases are self-generated in the process rather than the formation of BN and BC MAX phases, and more investigations need to be conducted in this context.

## 2. Outline of methodology

### 2.1 Materials

This research is focused on enhancing the semiconductor properties of MAX phase reinforced boron composites. The combination of the Ti_3_AlC_2_ or Ti_4_AIN_3_ MAX phase with amorphous boron can offer several advantages for semiconductor applications. The unique properties of MAX phases and amorphous boron can complement each other to provide a material with tailored characteristics suitable for semiconductor devices. Incorporating boron as amorphous boron (a-B) into these MAX phases further enhances their performance, particularly in semiconductor applications where materials with tailored electrical conductivity, and thermal and chemical stability are crucial. Amorphous boron is a promising material with unique properties including high hardness, excellent wear resistance, and interesting electrical and electronic properties.^17,18^ However, synthesizing pure, dense a-B-based MAX phase composites is challenging due to their complex structure and high melting point. Necessary materials and chemicals used in this research work for the synthesis of intended MAX phase composites were in the powder form. These materials were purchased from different sources. Among these, titanium (Ti) powder (Thermo Scientific, −325 mesh, 99.5% purity, CAS # 7440-32-6), aluminum (Al) powder (Thermo Scientific, −100 + 325 mesh, 99.5% purity, CAS # 7429-90-5), and boron nitride (BN) (Sigma-Aldrich, 99% purity, particle size: ≤150 nm, CAS # 10043-11-5) were purchased from USA. Other important chemicals like boron carbide (B_4_C) (Sigma-Aldrich, 98% purity, particle size: −200 mesh, CAS # 12069-32-8) and acetone (Merk KGaA, K42588114 838 1.00014.1000), utilized for this research work, were purchased from Germany. All other related materials like flash dry silver paint and thinner for flash dry silver paint (SPi supplies, USA, Lot no.: 1180905), and tungsten carbide (WC) powder (Inframat Advanced Materials, 99.9%, Lot: IAM7020WC6, Catalog: 74R-0606) were collected from the local market and utilized without additional purification.

### 2.2 Mass ratio calculation of mixture compounds

This calculation involves balancing the equation, converting substances into moles, using moles rate to estimate the moles of yielded substances in the reaction and converting the moles of required substances to the required units. Molar mass ratios of Ti, Al, BN and B_4_C for the preparation of working samples are shown in Table 1.

**Table 1:** Molar mass ratio calculations for the preparation of working samples

Equation	4Ti + Al + 2BN → Ti_4_AlB_2_N + N
Mass ratio	0.9576 : 0.1349 : 0.2482
Equation	4Ti + Al + B_4_C → Ti_4_AlB_2_C + 2B
Mass ratio	1.9132 : 0.2728 : 0.5523

### 2.3 Preparation of working samples (green samples)

**Fig. 1:**
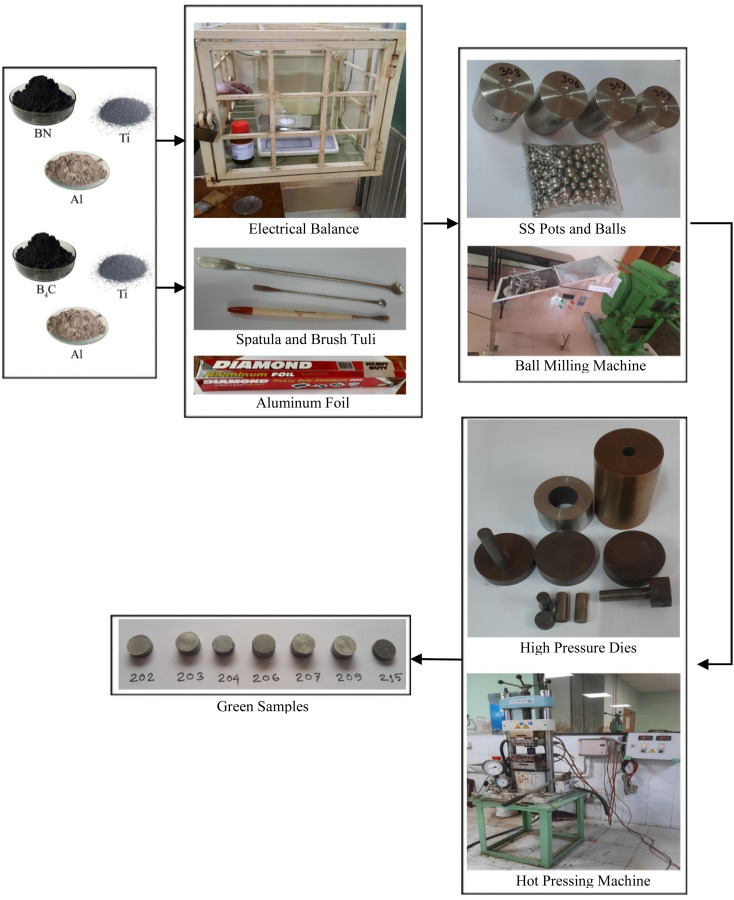
Synthesis process of green samples.

In this research work, the inert sintering process was used after mechanical alloying (ball milling) and hot pressing. A digital electronic balance machine (Model no.: GF12E US-1210, China), spatula (China), brush tuli (China) and aluminum foil (Diamond, China) were utilized for the measuring and weighing purpose of the materials. The pictorial flow diagram of the synthesis process of working samples is illustrated in Fig. 1.

Various powder materials (Ti, Al and BN or Ti, Al and B_4_C) were mixed in a planetary vertical ball milling machine applying a 5 : 1 sample and ball ratio (in weight) at 400 rpm for 4 h for the highest degree of fineness and mixing. A hydraulic hot press (PW 40H, Germany) was used to compress the powder materials at 30 MPa pressure and 380 °C temperature with a high pressure die and mold for obtaining the desired shape of Ti_4_AlN_3_ and Ti_3_AlC_2_ MAX phase reinforced boron composites (green samples). Initially, 15 MPa cold press was applied at ambient temperature, then the temperature was increased up to 380 °C, the pressure was increased up to 30 MPa gradually and held for 5 minutes to prepare the green samples. A total of 2 types of ten samples, 5 samples of Ti_4_AlB_2_N and 5 samples of Ti_4_AlB_2_C, were prepared and sintered at different temperatures to be used in experiments as shown in Table 2.

**Table 2:** Samples of Ti_4_AlB_2_N and Ti_3_AlB_2_C sintered at different temperatures

Sample compound	Samples' names and their sintering temperatures				
	950 °C	1050 °C	1150 °C	1250 °C	1325 °C
Ti_4_AlB_2_N	Sample 306C	Sample 206B	Sample 306A	Sample 306B	Sample 206A
Ti_4_AlB_2_C	Sample 309C	Sample 209B	Sample 309A	Sample 309B	Sample 209A

**Fig. 2:**
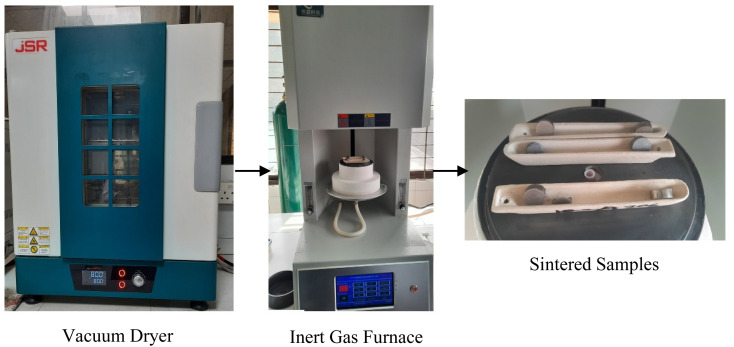
Vacuum drying and inert gas sintering process.

The green samples were inserted into a Vacuum Dryer (JSVO-30T, Korea) for 24 hours at 80 °C before sintering. The dried samples were taken into an inert gas furnace (Dental elevator, Model: CY-1400-cocr, China) and sintered at 950 °C, 1050 °C, 1150 °C, 1250 °C and 1325 °C temperatures in a porcelain vessel with a heating and cooling rate of 5 °C min^−1^ with 2 hours holding time for each sample. The inert environment in the furnace was maintained using Ar gas with a mass flow rate of 4–5 L min^−1^. The overall drying and sintering process of the green samples is shown in Fig. 2.

**Fig. 3:**
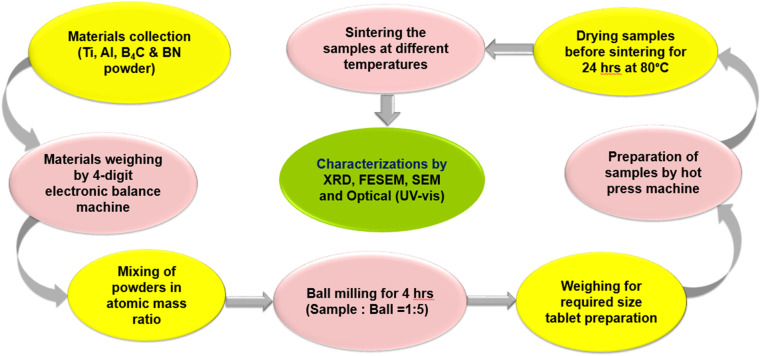
Schematic flow diagram of the overall synthesis process and experimental.

After cooling and necessary preparations, the sample composites were used for further testing and analysis. The schematic flow diagram of the overall synthesis process and experimental techniques is shown in Fig. 3.

Molar mass ratios of powder compositions, sintering method, sintering temperature, sintering time, inert environment, retarding of oxide formation (drying), and also diffusion were taken into account in this study. Their effects on structural and phase formation were investigated.

Characteristics and phase formation were analyzed by using different characterization techniques as mentioned below.

## 3. Characterization techniques

### 3.1 XRD

A Malvern Panalytical instrument, Netherlands, Model: 9430 060 03000 was used for XRD analysis. Data were accumulated by a using data viewer with “ORIGIN” and then “HIGH SCORE” software. Measurement conditions of scan range: 20° to 89°, configuration: flat sample stage, goniometer: theta/theta, minimum step size 2theta: 0.0001, minimum step size omega: 0.0001, sample stage: stage for flat samples/holders, diffractometer system: EMPYREAN, anode material: Cu, K-α1 wavelength: 1.540598 Å, divergence slit type: fixed and slit size [°]: 0.76, generator voltage: 45 kV, tube current: 40 mA, *h k l*: 0 0 0, scan axis: Gonio, scan step size [°2*θ*]: 0.0262606, number of points: 3046, scan type: continuous, time per step (s): 46.665 were considered. The MAX phase composite samples were first ground into fine powders using a mortar and pestle to confirm uniform particle size distribution. The powder was then placed into a sample holder. Finally, the sample was carefully aligned in the XRD instrument to ensure accurate diffraction measurements across the desired range of 2*θ* angles.

### 3.2 FESEM

A field emission scanning electron microscopy (FESEM) instrument of Model: JSM-7610F JEOL, Japan was used to get topographical data on the surface of fabricated sample composites using PC-SEM, JEOL software. The operating conditions of volt: 15 kV, probe current: 1 nA, PHA mode: T3, counting rate: 77 cps, energy range: 0–20 keV, method: ZAF and fitting coefficient: 0.6414 were applied to get high magnification images of the samples for necessary analysis. For the FESEM tests, sintered tablet samples were cleaned with an isopropanol to remove any debris and gold coated by an agar sputter coater, and then the sample was placed in the test chamber at an appropriate accelerating voltage to capture high-resolution images of the microstructure.

### 3.3 SEM

A scanning electron microscopy (SEM) machine of TESCAN, Czech Republic, Model: VEGA COMPACT LMH 123-0141 was used for SEM analysis of targeted sample surfaces. The operating conditions of mode: continual, dead time: 2%, landing energy: 15 keV, beam current: 3 nA and coating element: gold were applied to conduct this test.

### 3.4 Optical (UV-vis)

A UV-vis spectrophotometer of Model: A12536103565 CD, SHIMADZU, UV-1900i, China was used for optical characterization of synthesized MAX phase composite samples. The machine was calibrated first and then the sample holder and standard vial were set. The linear and non-linear absorbance of 0–1 Au was fixed. The wavelength (*λ*) range 190–1100 nm was taken and the sample was considered as the direct material where *n* = ½ was set.

## 4. Results and discussion

### 4.1 XRD analysis

**Fig. 4:**
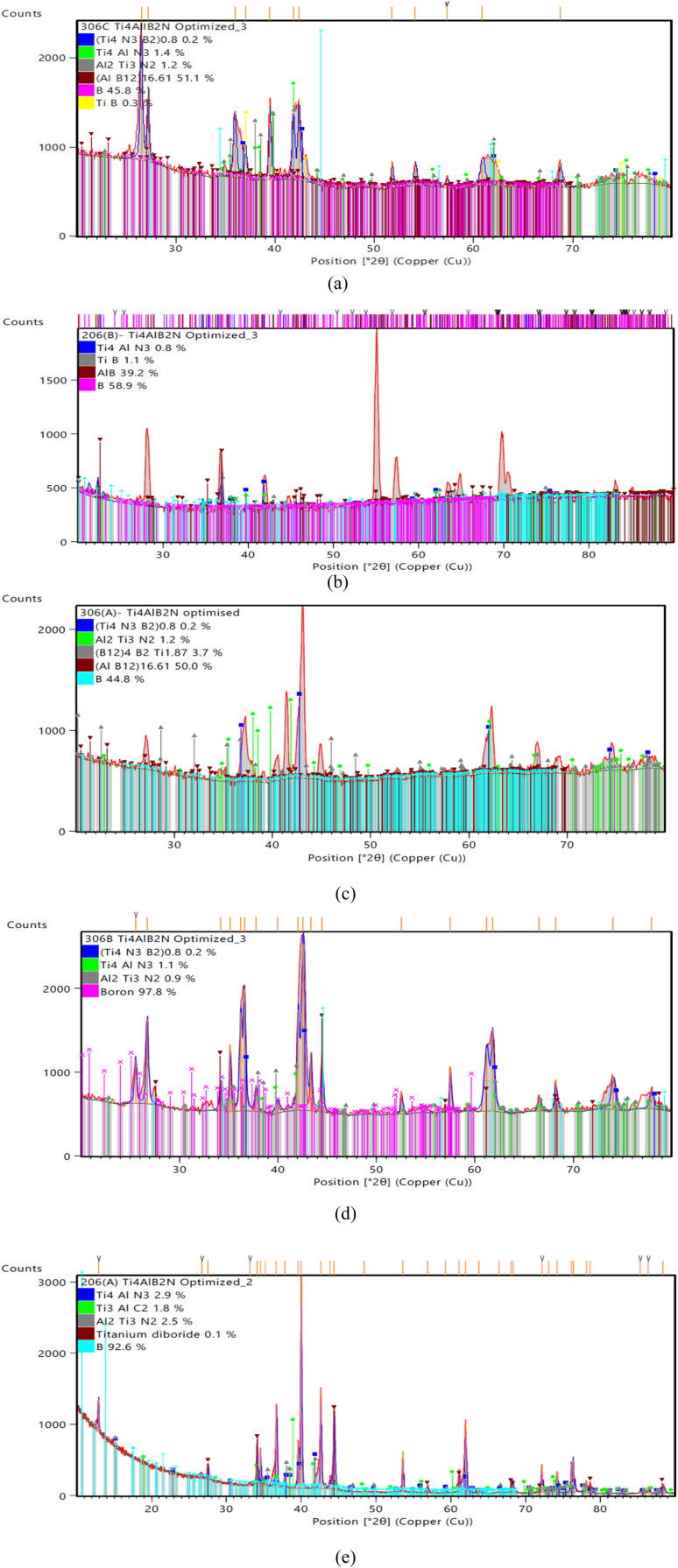
Main graphics analysing the view of XRD patterns of Ti_4_AlB_2_N samples sintered at different temperatures: (a) sample sintered at 950 °C, (b) sample sintered at 1050 °C, (c) sample sintered at 1150 °C, (d) sample sintered at 1250 °C and (e) sample sintered at 1325 °C.

**Fig. 5:**
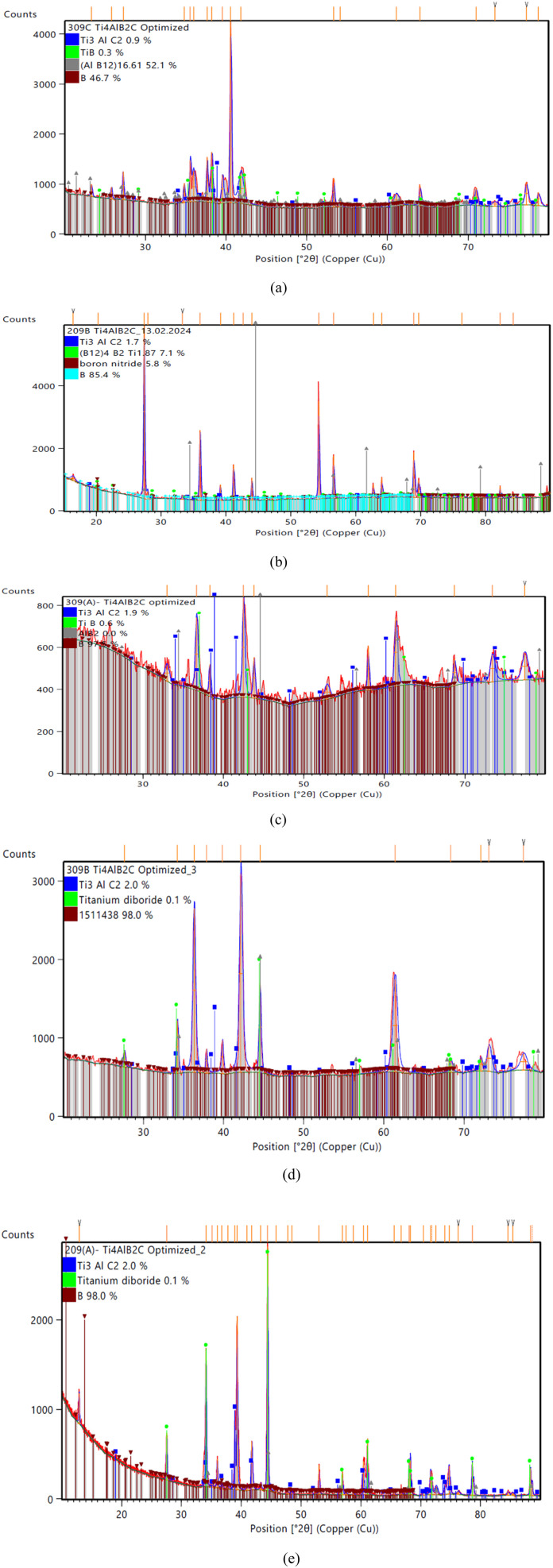
Main graphics analysing the view of XRD patterns of Ti_4_AlB_2_C samples sintered at different temperatures: (a) sample sintered at 950 °C, (b) sample sintered at 1050 °C, (c) sample sintered at 1150 °C, (d) sample sintered at 1250 °C and (e) sample sintered at 1325 °C.

**Fig. 6:**
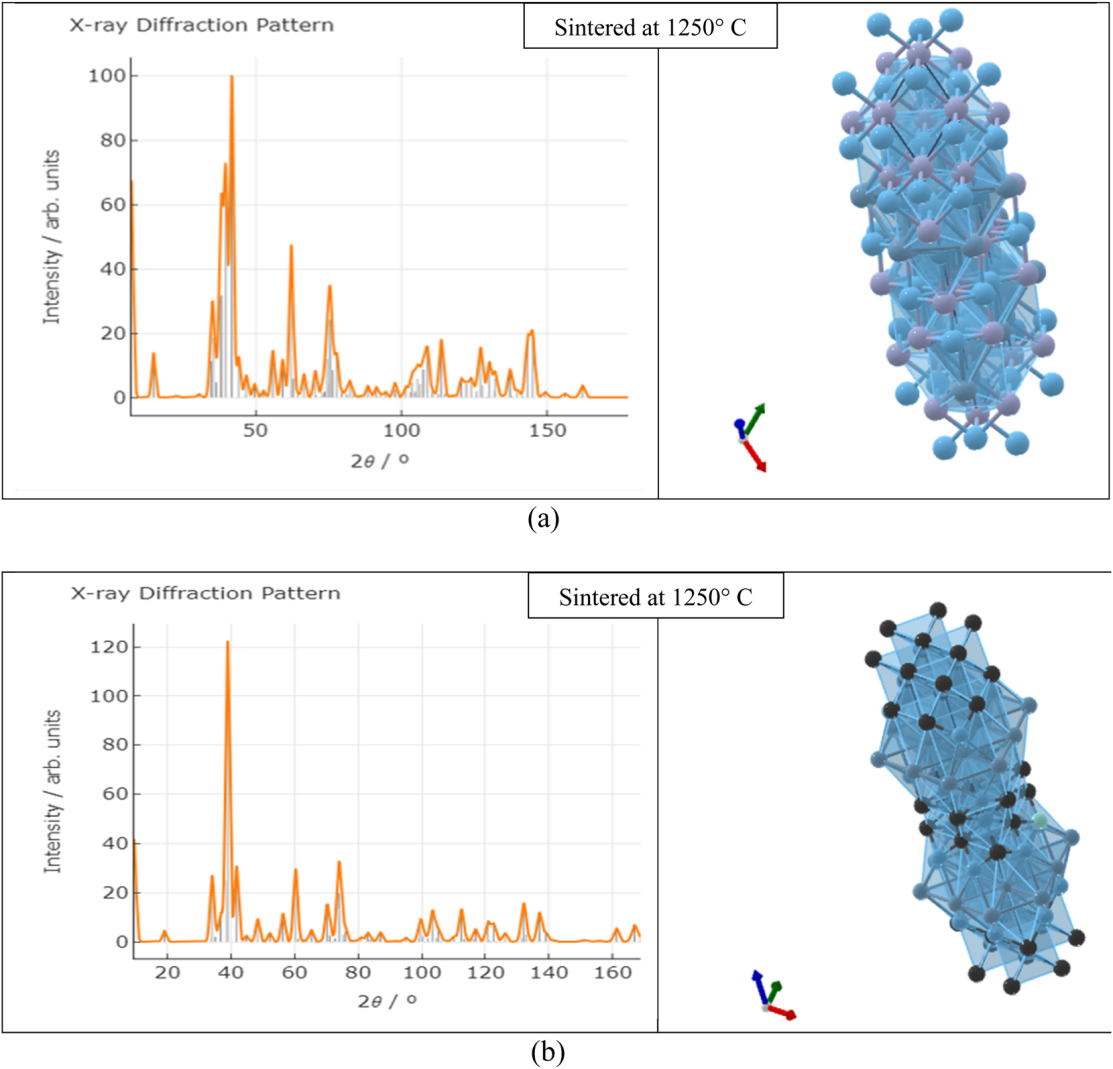
X-ray diffraction pattern and crystal structure (*P*6_3_/*mmc*) of (a) Ti_4_AlN_3_ and (b) Ti_3_AlC_2_ MAX phases.

The specimen under investigation was in a highly pulverized form, having undergone extensive mixing to ensure uniformity. The sintered samples as prepared were scanned through the 2*θ* angle range to capture all lattice scattering orientations caused by random powder material orientations.^23,24^ In order to examine the development of Ti_3_AlC_2_ and Ti_4_AlN_3_ MAX phases and amorphous boron and determine the interplanar distance in green samples, XRD experiments were conducted. The peak analyzing the view of main graphics of all XRD spectra shows the percentage of various compounds in fabricated composites that were sintered at different temperatures, which are depicted in Fig. 4 and 5 and projected in Table 3. From Table 3, it is found that in both the cases the MAX phase is formed as shown in Fig. 6(a) and (b), which is usually increased slightly from 0.2% to 2.9% with increasing temperatures from 950 °C to 1325 °C in the case of Ti_4_AlB_2_N samples. With increasing temperature, it is also found that the percentage of boron is increased from 45.8% to 97.8%. Again, in the case of Ti_4_AlB_2_C samples, the MAX phase is found to increase from 0.9% to 2.0% and the percentage of boron is found to increase from 46.7% to 98.0% under similar conditions. With increasing temperature and particularly the onset of over-sintering it is also observed that additional phases or intergranular reactions occur, reintroducing defects and potentially forming secondary phases in both the cases for all the fabricated sample composites.

**Table 3:** Summary of compositions found from the XRD results of Ti_4_AlB_2_N and Ti_4_AlB_2_C samples

Sample compound	Compositions of samples sintered at different temperatures					Remarks
Ti_4_AlB_2_N	Sample 306C (950 °C)	Sample 206B (1050 °C)	Sample 306A (1150 °C)	Sample 306B (1250 °C)	Sample 206A (1325 °C)	MAX phase formation increases with the increase of sintering temperature. Amorphous B is dispersed uniformly
	Ti_4_AlN_3_ = 1.4%, (Ti_4_N_3_B_2_) = 0.2%, Al_2_Ti_3_N_2_ = 1.2%, (AlB12) = 51.1%, B = 45.8%, TiB = 0.3%	Ti_4_AlN_3_ = 0.8%, TiB = 1.1%, AlB = 39.2%, B = 58.9%	Ti_4_AlN_3_ = 0% (Ti_4_N_3_B_2_) = 0.2%, Al_2_Ti_3_N_2_ = 1.2% (B12)_4_B_2_Ti = 3.7%, (AlB12) = 50.0%, B = 44.8%	Ti_4_ AlN_3_ = 1.1% (Ti_4_N_3_B_2_) = 0.2%, Al_2_Ti_3_N_2_ = 0.9%, B = 97.8%	Ti_4_AlN_3_ = 2.9%, Ti_3_AlC_2_ = 1.8%, Al_2_Ti_3_N_2_ = 2.5%, TiB_2_ = 0.1%, B = 92.6%	
Ti_4_AlB_2_C	Sample 306C (950 °C)	Sample 206B (1050 °C)	Sample 306A (1150 °C)	Sample 306B (1250 °C)	Sample 206A (1325 °C)	MAX phase formation increases with the increase of sintering temperature. Amorphous B is dispersed more uniformly
	Ti_3_AlC_2_ = 0.9%, TiB = 0.3%, (AlB12) = 52.1%, B = 46.7%	Ti_3_AlC_2_ = 1.7%, (B12)_4_B_2_Ti = 7.1%, BN = 5.8%, B = 85.4%	Ti_3_AlC_2_ = 1.9%, TiB = 0.6%, AlB_2_ = 0.0%, B = 97.5%	Ti_3_AlC_2_ = 2.0%, TiB_2_ = 0.1%, B = 98.0%	Ti_3_AlC_2_ = 2.0%, TiB_2_ = 0.1%, B = 98.0%	

#### 4.1.1 Crystallinity

Crystallinity refers to the extent to which a material is composed of ordered arrangements of atoms or molecules in a crystalline lattice structure, resulting in distinct properties such as a regular shape, sharp melting point, and high mechanical strength. The degree of crystallinity of fabricated composites is calculated by using the formula mentioned in eqn (1).^25^ The degree of crystallinity (%),1
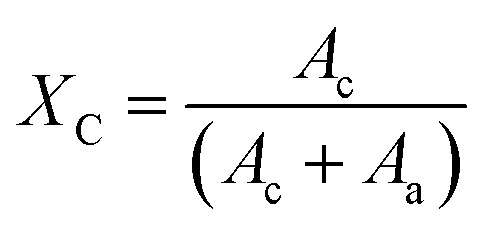
where *A*_c_ = crystalline area, and *A*_a_ = amorphous area.

**Fig. 7:**
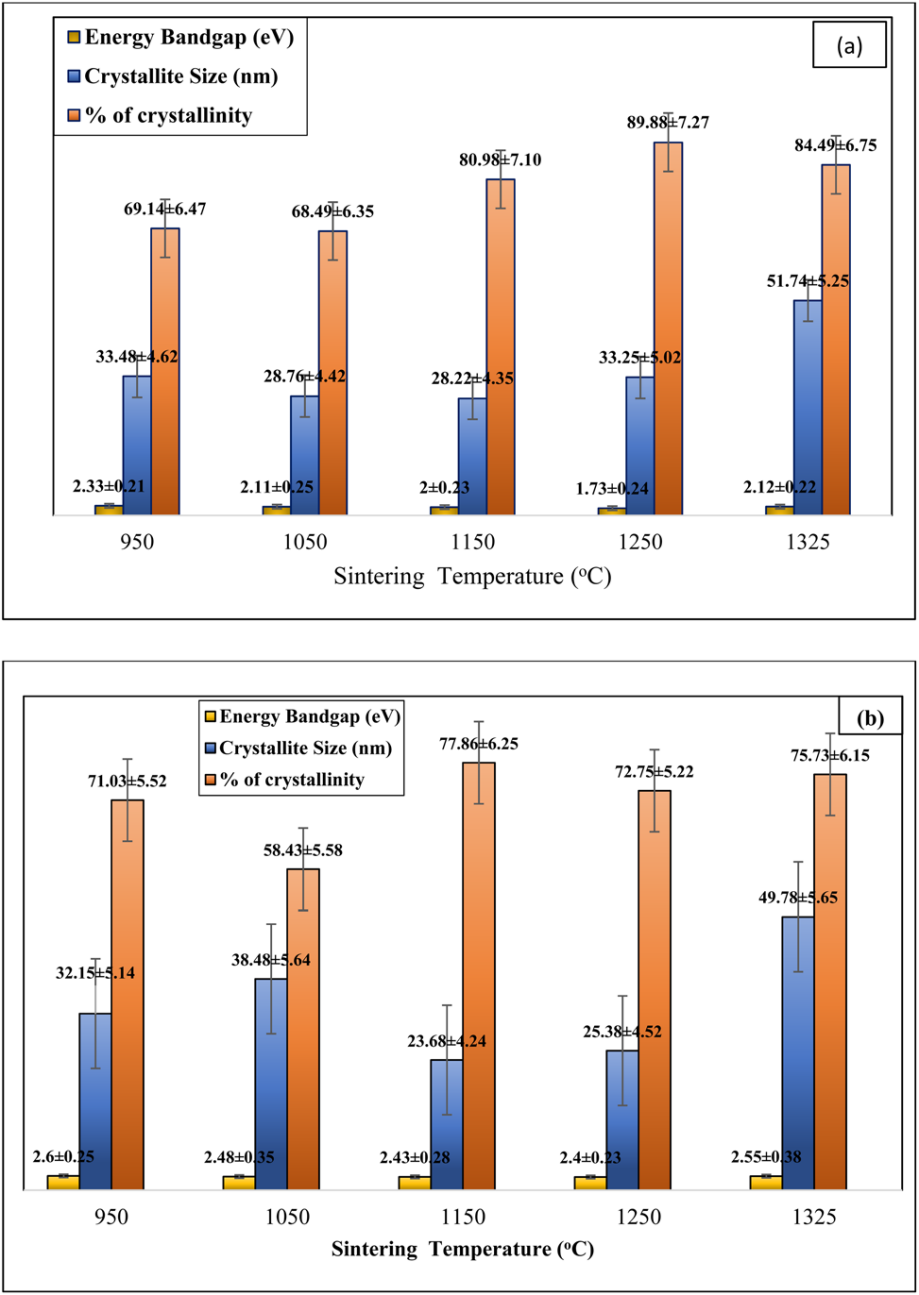
Relation of % of crystallinity and crystallite size with energy bandgaps of fabricated boron based *in situ* MAX phase reinforced composites. (a) Ti_4_AlB_2_N (*in situ* Ti_4_AlN_3_ MAX phase reinforced composite) and (b) Ti_4_AlB_2_C (*in situ* Ti_3_AlC_2_ MAX phase reinforced composites).

From Table 4 and Fig. 7, it is observed that the percentage of crystallinity increases for both the composites with increasing sintering temperature. In the case of Ti_4_AlN_3_ MAX phase reinforced boron-based composites, the percentage of crystallinity increases from 69.14% to 89.88% and in the case of Ti_3_AlC_2_ MAX phase reinforced boron based composites the percentage of crystallinity increases from 71.02% to 77.86%. And it is observed that when the percentage of crystallinity increases, the energy bandgap shows a declining trend from 2.33 eV to 1.78 eV for Ti_4_AlB_2_N sample composites and 2.60 eV to 2.40 for Ti_4_Al B_2_C sample composites. Higher crystallinity generally enhances the mechanical, thermal, and optical properties of a material. In highly crystalline materials, atoms are arranged in a regular, repeating pattern, which facilitates efficient transmission of electrons and phonons, leading to better electrical conductivity and thermal conductivity. Additionally, high crystallinity often results in increased strength and hardness because the orderly atomic structure can better resist deformation. Conversely, materials with low crystallinity, such as amorphous materials, typically exhibit lower mechanical strength and thermal conductivity but may have superior flexibility and impact resistance.^26^

**Table 4:** Experimental results of energy bandgaps, % of crystallinity, average crystallite size and average micro strain of fabricated Ti_4_AlB_2_N and Ti_4_AlB_2_C sample composites

Sample name	Sintering temperature (°C)	Energy bandgap (eV)	% of crystallinity	Average crystallite size, *D* (nm)	Average dislocation density	Average microstrain, (*ε*) *δ*
Ti_4_AlB_2_N	950	2.33	69.14479301	33.4801158	0.000892126	0.174
	1050	2.11	68.49341763	28.75657405	0.001209277	0.3706
	1150	2.00	80.98498124	28.22378443	0.001255364	0.1905
	1250	1.73	89.87724096	33.25336288	0.000904334	0.1809
	1325	2.12	84.49385884	51.74103091	0.000373534	0.13325
Ti_4_AlB_2_C	950	2.60	71.02942065	32.14693379	0.000967656	0.1954
	1050	2.48	58.43002545	38.47868919	0.000675398	0.214
	1150	2.43	77.85832197	23.68582858	0.001782472	0.2405
	1250	2.40	72.7525578	25.37922385	0.001552542	0.2148
	1325	2.55	75.72842043	49.78197252	0.000403511	0.1309

#### 4.1.2 Crystallite size

Crystallite size, or grain size, is a measure of the size of coherently diffracting domains within a material. The crystallite size (nm) of fabricated composites is calculated by using the Scherrer equation as mentioned in eqn (2).^27,28^2
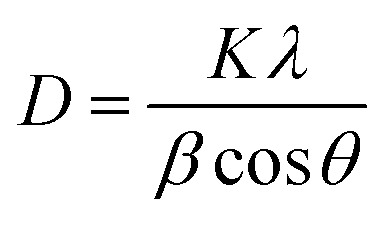
where *K* = 0.9 (Scherrer constant), *λ* = 0.154006 nm (wavelength of the X-ray source), *β* = FWHM (full width at half maximum) in radians, and *θ* = peak position (radians).

From the tabulated results in Table 4 and Fig. 7, it is observed that there is a small variation noticed in the crystallite size of fabricated samples with increasing temperature from 950 °C to 1250 °C and an optimal crystallite size is found in this range but after 1250 °C, the variation in crystallite size was observed to be more. Smaller crystallites, or nanocrystallites, can enhance certain properties, such as hardness and strength, through the Hall–Petch effect, which states that smaller grains inhibit dislocation motion, thereby strengthening the material. However, very small grains can also lead to an increased grain boundary area, which can act as sites for corrosion and reduce the material's overall ductility. Larger crystallites usually mean fewer grain boundaries, which can enhance the material's ductility and electrical conductivity but may reduce hardness and strength. The optimal crystallite size is often a balance between these competing effects and depends on the intended application of the material.^29^

#### 4.1.3 Dislocation density

Dislocation density is the measure of the number of dislocations in a unit volume of a material. The dislocation density of all the fabricated composites is calculated by eqn (3):^30^

3 Dislocation density, *δ* = 1/*D*^2^

where *D* = crystallite size (nm).

From the calculated results as shown in Table 4 and projected in Fig. 7, it is found that there is a declining trend in average dislocation density from 0.0012 to 0.0003 with increasing sintering temperature in the case of Ti_4_AlN_3_ MAX phase reinforced boron-based composites, and in the case of Ti_3_AlC_2_ MAX phase reinforced boron-based composites it is observed to be from 0.0009 to 0.0004. In both the cases the average dislocation density is found to be very low and well controlled. Low dislocation density is generally desired for applications requiring high ductility and toughness, as it reduces internal stresses and potential sites for crack initiation. High dislocation density typically results from plastic deformation or rapid solidification processes and can significantly impact a material's mechanical properties. While dislocations can strengthen a material by impeding the movement of other dislocations (work hardening), excessive dislocations can also act as stress concentrators, leading to increased brittleness and a higher likelihood of fracture. In materials where high strength is crucial, controlling dislocation density is essential, as it directly influences the yield strength and fatigue resistance.^31,32^

#### 4.1.4 Microstrain

Microstrain refers to the distribution of small elastic strains within a material, typically caused by defects, dislocations, or variations in lattice parameters. Microstrain can affect a material's diffraction patterns and lead to broadening of X-ray diffraction peaks, providing insights into the internal stresses and imperfections of the material. The microstrain of all the fabricated composites is found out with the help of eqn (4) (ref. 33) below:

4 Microstrain, *ε* = *β*/4 tan *θ*

where *β* = FWHM (radians), and *θ* = peak position (radians).

From the calculation it is found that the average microstrain is reduced from 0.370 to 0.133 in the case of Ti_4_AlB_2_N samples and 0.195 to 0.130 in the case of Ti_4_AlB_2_C samples. Managing microstrain is crucial in materials design, especially for high-performance applications, as it ensures structural integrity and reliability.^34^ High microstrain often indicates a high level of internal stress, which can lead to premature failure under mechanical loading. It can also impact the material's mechanical properties, including its yield strength and fatigue life. However, microstrain can be reduced through processes like annealing, which can enhance ductility and toughness by relieving internal stresses.

### 4.2 FESEM analysis

**Fig. 8:**
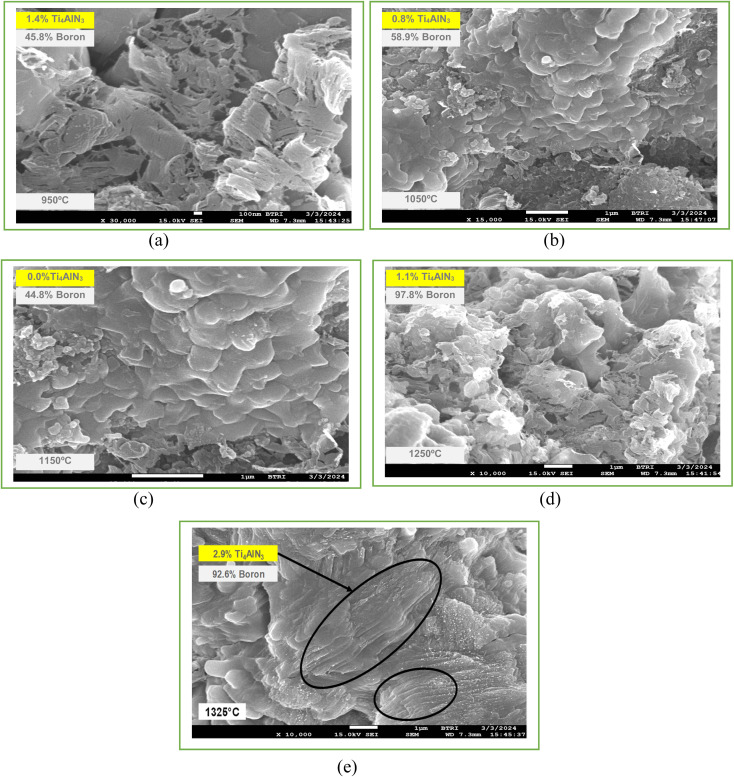
FESEM images of the Ti_4_AlB_2_N composite (a) sample sintered at 950 °C, (b) sample sintered at 1050 °C, (c) sample sintered at 1150 °C, (d) sample sintered at 1250 °C and (e) sample sintered at 1325 °C.

**Fig. 9:**
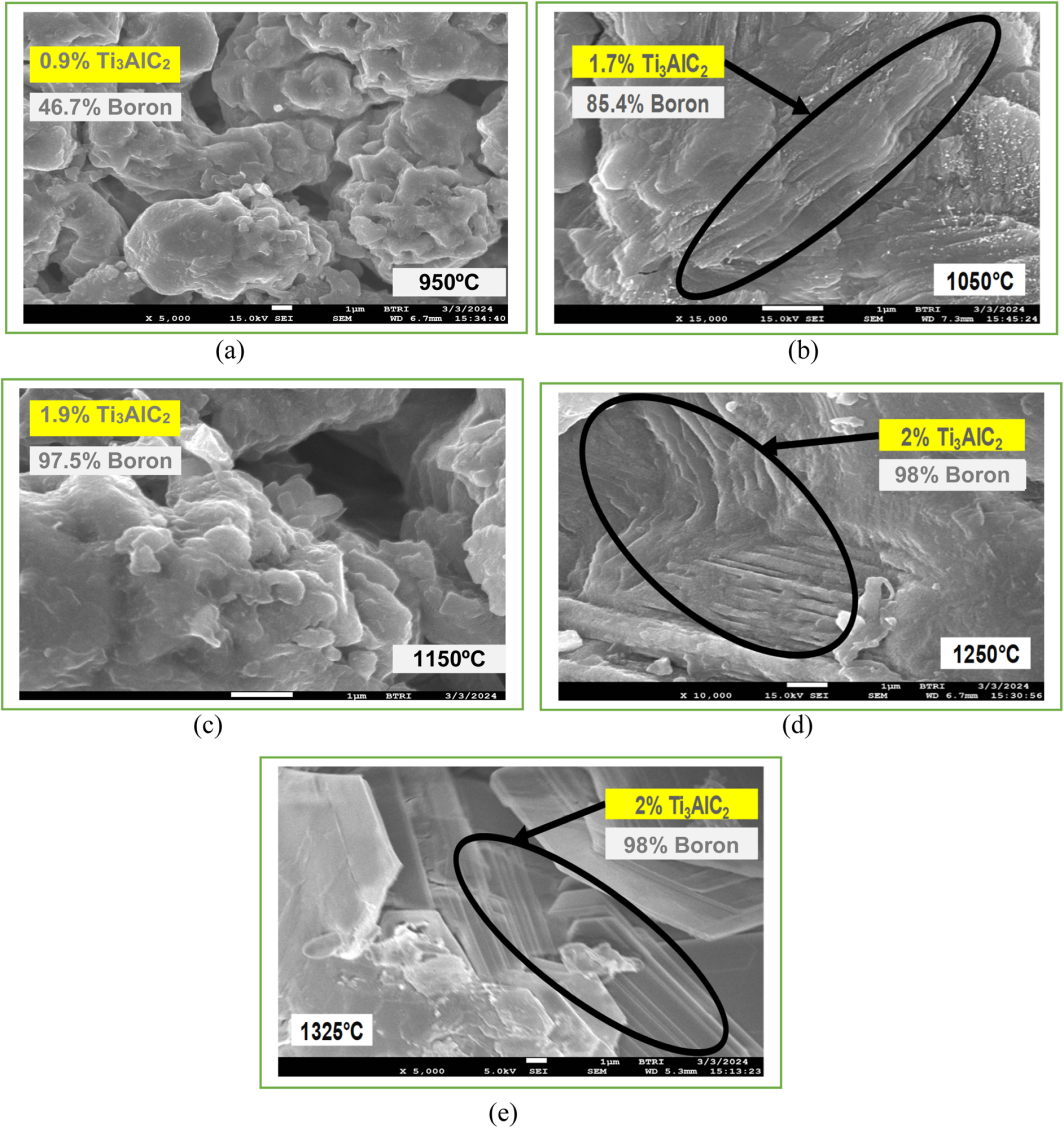
FESEM images of the Ti_3_AlB_2_C composite (a) sample sintered at 950 °C, (b) sample sintered at 1050 °C, (c) sample sintered at 1150 °C, (d) sample sintered at 1250 °C and (e) sample sintered at 1325 °C.

Field Emission Scanning Electron Microscopy (FESEM) provides detailed images of the surface morphology revealing characteristic layered structures. These layered structures are often indicative of the presence of MAX phases. While FESEM provides surface and microstructural details confirming MAX phase formation, it often requires a correlation with XRD, which gives definitive information about the crystal structure.^35–38^ By correlating the high-resolution images and elemental composition data from FESEM (as shown in Fig. 8 and 9) with the crystal structure information from XRD (as shown in Fig. 6 and Table 4), the formation of MAX phases can be robustly confirmed. The detailed surface and compositional analyses from FESEM support and verify the structural findings from XRD results as shown in Table 4, providing a comprehensive understanding of the material's properties and confirming the successful synthesis of the desired MAX phase composites.

### 4.3 SEM analysis

**Fig. 10:**
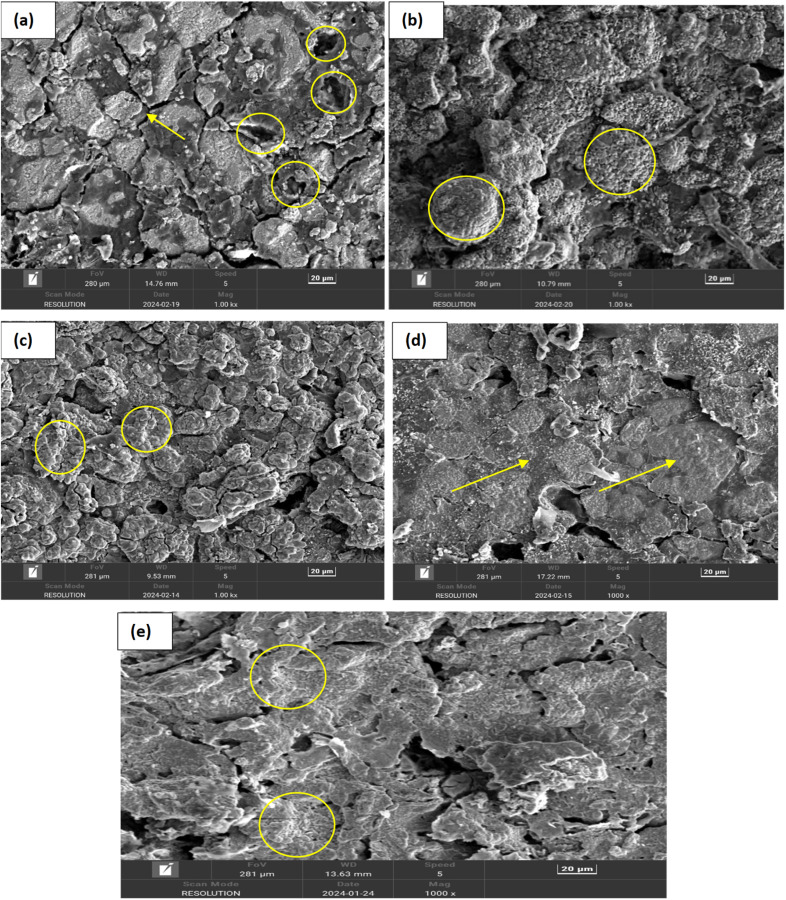
SEM images of Ti_4_AlB_2_N composites at different sintering temperatures: (a) 950 °C, the arrow and circle indicate voids and cracking of the composite surface; (b) 1050 °C, the circle indicates grain boundary development; (c) 1150 °C, the marks indicate the development of grain growth and agglomeration of particles; (d) 1250 °C, the marks show a denser packing structure; and (e) 1325 °C, the corresponding marks indicate a highly dense and well-sintered composite with minimal porosity and large, well-bonded grains with few MAX phase layers.

**Fig. 11:**
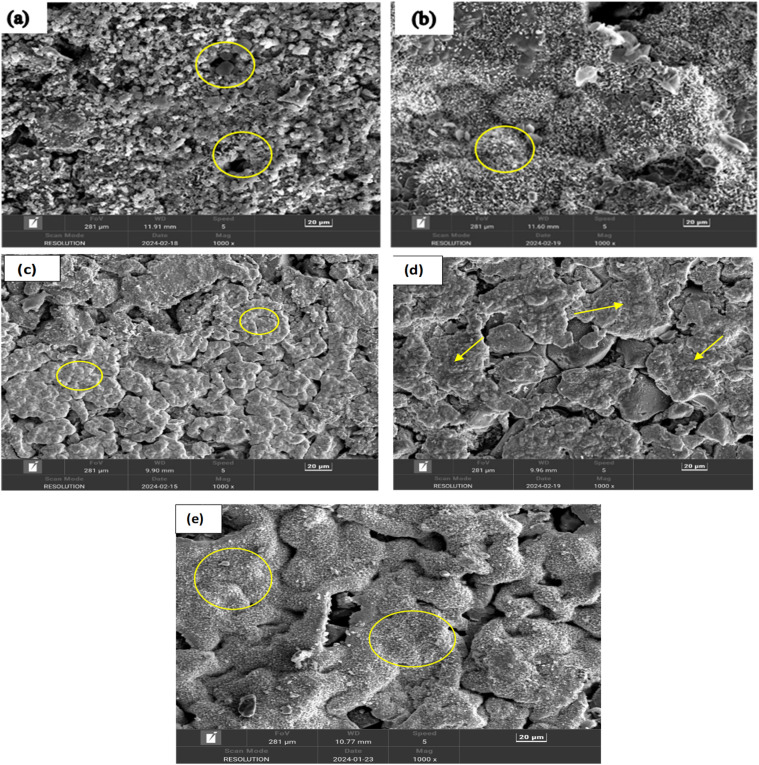
The SEM images of Ti_3_AlB_2_C composites at varying sintering temperatures. (a) 950 °C, the corresponding marks indicate voids and loosely bonded particles on the composite surface; (b) 1050 °C, the circle indicates that grain boundaries are developed; (c) 1150 °C, the consistent symbols indicate grain growth and agglomeration of particles; (d) 1250 °C, the arrow shows a denser packing structure growth on the surfaces; and (e) 1325 °C, the corresponding marks specify a highly dense and well-sintered composite with minimal porosity and well-bonded grains with amorphous boron.

In order to investigate the surface morphology of self-generated Ti_4_AlN_3_ and Ti_3_AlC_2_ MAX phase composites with amorphous boron, the SEM test was conducted. The effect of sintering temperature on the microstructure of MAX-phase composites was observed through this investigation. Fig. 10(a–e) and 11(a–e) display the SEM images of various MAX-phase composites sintered at different temperatures (950 °C, 1050 °C, 1150 °C, 1250 °C and 1325 °C). At 950 °C, the composite shows incomplete sintering with noticeable porosity and loosely bonded grains, indicating limited diffusion and phase formation, as seen for both Ti_4_AlB_2_N and Ti_3_AlB_2_C composites in Fig. 10 (a) and 11(a). At this temperature, the SEM images also show intergranular cracks and voids throughout the surfaces of MAX phase composites. As the temperature increases to 1050 °C, the images reveal improved grain bonding and reduced porosity, suggesting enhanced diffusion and initial densification (Fig. 10(b) and 11b). The grains become very large and the boundaries become less distinct, indicating that the material has undergone significant reorganization, which can enhance its phase stability and impact its properties.^39^

For sintering temperature at 1150 °C, the microstructure exhibits more pronounced grain growth and a denser packing, with fewer voids and better-defined grain boundaries, indicating significant phase formation and consolidation. Moreover, the agglomerates of chemical compositions are also observed in Fig. 10(c) and 11(c) at this temperature. Fig. 10(d) and 11(d) depict a more compact surface structure for both Ti_4_AlB_2_N and Ti_3_AlB_2_C composites sintered at a temperature of 1250 °C. This indicates the successful formation of Ti_4_AlN_3_ and Ti_3_AlC_2_ MAX phases with amorphous boron, as observed in other studies.^40–43^

Finally, at 1325 °C, the SEM images display a highly dense and well-sintered composite, with minimal porosity and large well-bonded grains. At this temperature, the composite surfaces also showed some layers of Ti_4_AlN_3_ MAX phase (Fig. 10(e)) and Ti_3_AlC_2_ MAX phase (Fig. 11(e)). However, amorphous boron was more visible on Ti_3_AlC_2_ MAX phase composite surfaces than that of the Ti_4_AlN_3_ MAX phase. The amorphous boron regions become more integrated within the MAX matrix, contributing to a more uniform and homogeneous microstructure.^44–49^ These images collectively demonstrate the critical role of sintering temperature in influencing the densification, grain growth, and overall microstructural integrity of the MAX phase composites. Furthermore, the microstructural changes with temperature directly influence the composite's mechanical strength, toughness, semiconductor properties, and thermal stability which are important for potential applications. The amorphous boron is visible at dispersed regions within the matrix, often appearing more uniform and less structured due to its non-crystalline nature. As previously stated, all of these lead to the *in situ* formation of amorphous boron with dispersed Ti_4_AlN_3_ and Ti_3_AlC_2_ MAX phases in the composites.

### 4.4 Ultraviolet-visible (UV-vis) spectroscopy analysis

**Fig. 12:**
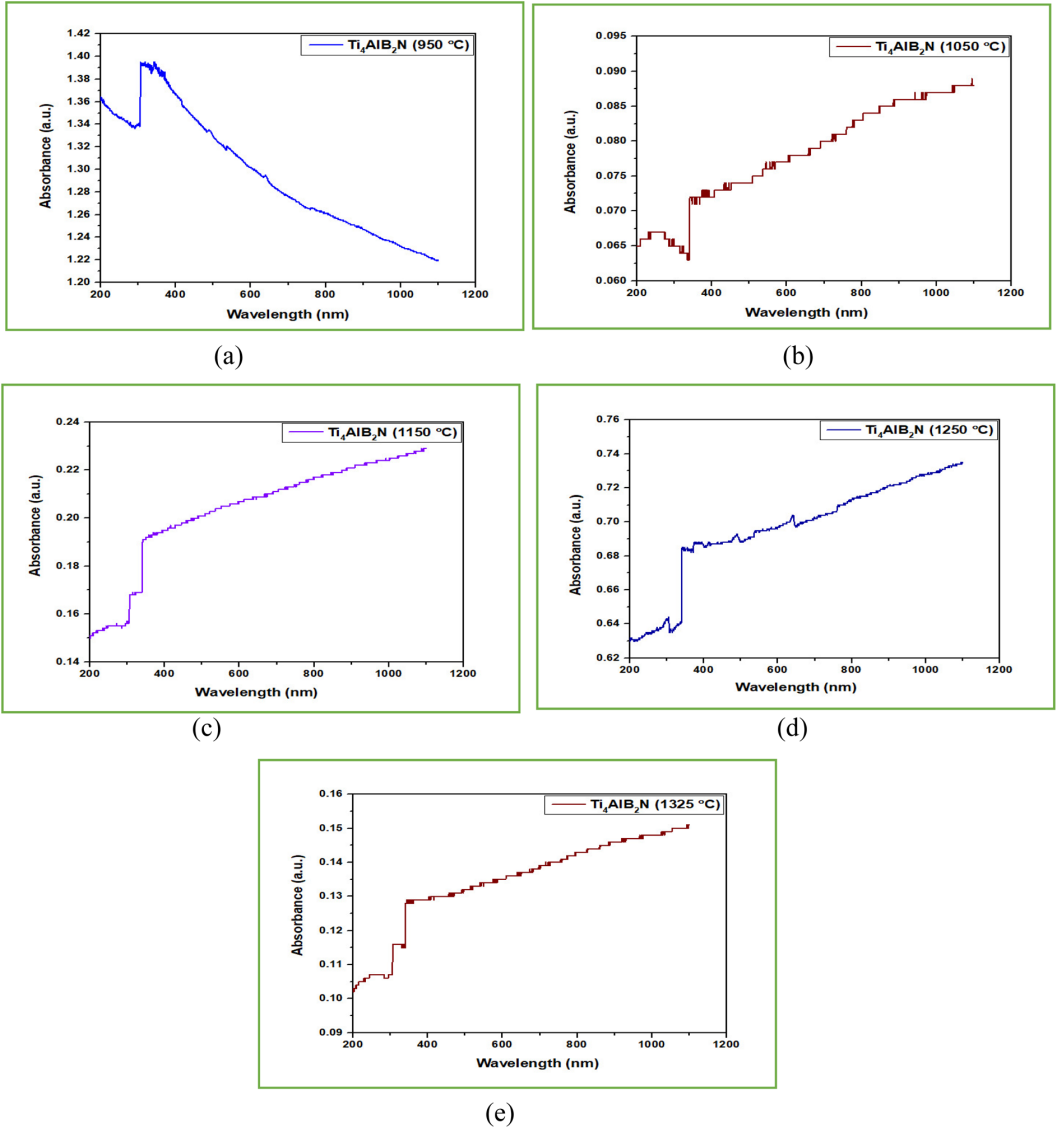
UV analysis of Ti_4_AlB_2_N (absorbance *vs.* wavelength): (a) sample sintered at 950 °C, (b) sample sintered at 1050 °C, (c) sample sintered at 1150 °C, (d) sample sintered at 1250 °C and (e) sample sintered at 1325 °C.

**Fig. 13:**
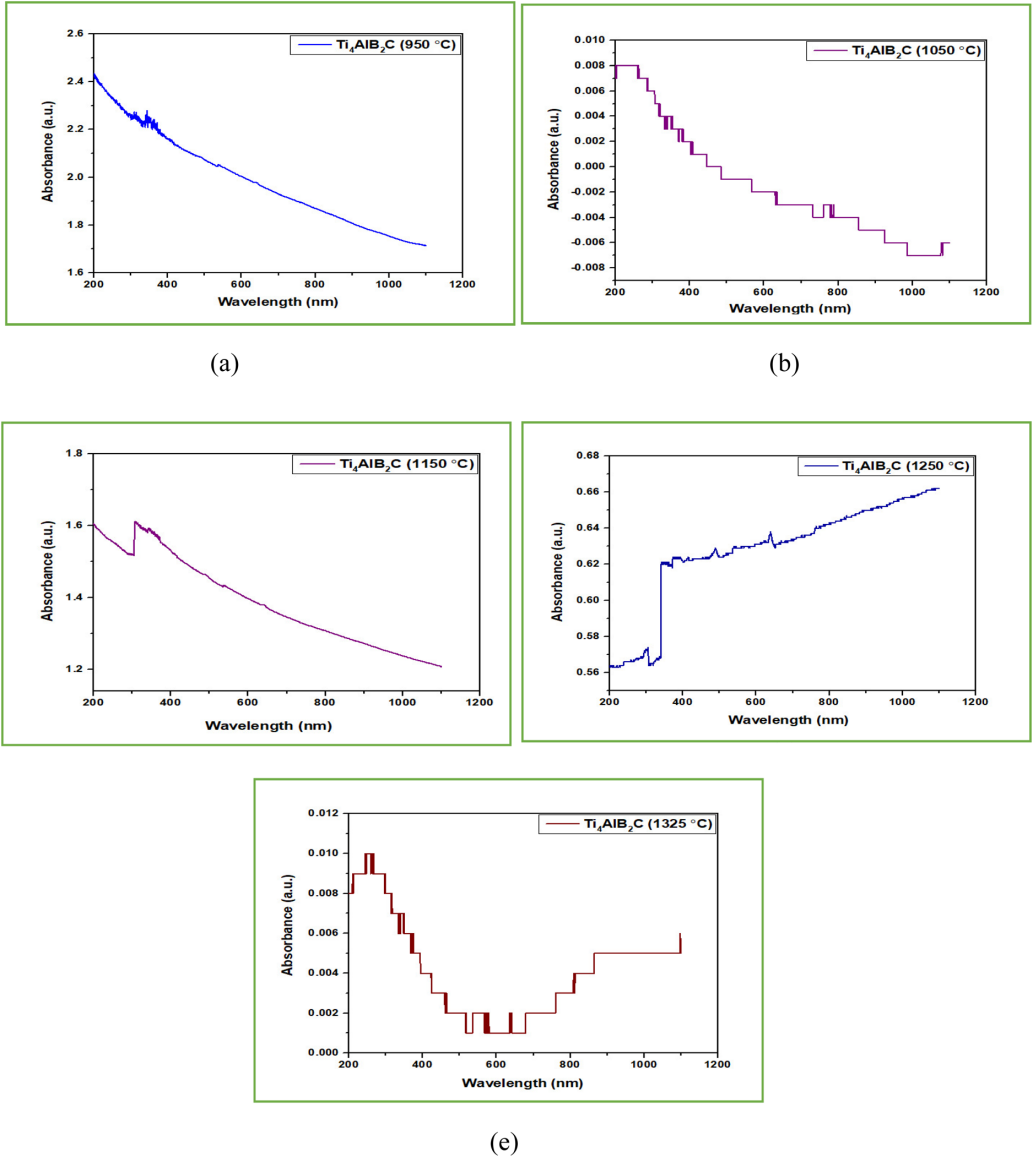
UV analysis of Ti_4_AlB_2_C (absorbance *vs.* wavelength): (a) sample sintered at 950 °C, (b) sample sintered at 1050 °C, (c) sample sintered at 1150 °C, (d) sample sintered at 1250 °C and (e) sample sintered at 1325 °C.

UV-vis spectroscopy refers to absorption spectroscopy or reflectance spectroscopy in part of the ultraviolet and the full, adjacent visible regions of the electromagnetic spectrum.^50^ UV-vis tests were conducted to understand the optical characteristics of different Ti_4_AlB_2_N and Ti_4_AlB_2_C samples. The UV spectra of all sintered samples are shown in Fig. 12 and 13.

#### 4.4.1 UV spectra analysis of Ti_4_AlN_3_ MAX phase reinforced boron based composites

In a UV (ultraviolet) test for a boron-based Ti_4_AlN_3_ MAX phase composite, the absorbance rate varies from 0.065 a.u. to 0.63 a.u. with increasing sintering temperature from 1050 °C to 1325 °C and in all these cases the absorbance rate increases with increasing wavelength. Absorbance in the range of 0.05 to 0.65 a.u. means that the material absorbs a controlled amount of UV radiation. This would generally be considered better for semiconductor applications. It also helps in protecting the semiconductor from excessive UV exposure, which can cause degradation, generation of defects, and changes in electronic properties over time. By avoiding high levels of UV absorption, the material retains its structural integrity and electronic performance. Moderate absorbance prevents excessive heating due to UV exposure. Excessive heating can lead to thermal stress, which negatively affects the material's mechanical and electrical properties. Maintaining absorbance within this range ensures that any heat generated is manageable, thus preserving the thermal stability of the semiconductor device.^51,52^

For optoelectronic devices such as photodetectors, solar cells, and LEDs, a balanced absorbance rate ensures that the material is not overly opaque, allowing for efficient light transmission and interaction with the active regions. This balance is crucial for optimizing the device's performance by enabling effective light absorption where needed without excessive thermal losses. An absorbance rate in this range helps in preventing the formation of UV-induced defects by not allowing too much UV energy to penetrate and interact with the semiconductor material. This helps in maintaining the purity and performance of the semiconductor, avoiding issues such as increased electron–hole recombination rates that can reduce device efficiency.^53,54^

High absorbance rates are generally detrimental to semiconductor applications due to the associated risks of material degradation, thermal instability, reduced optoelectronic performance, and increased susceptibility to contamination effects. Ensuring low to moderate UV absorbance (from 0.1 to 1.6 a.u.) helps maintain the structural and electronic integrity of semiconductor materials, thereby enhancing device reliability and efficiency. An absorbance rate in the range of 0.05 to 0.65 a.u. for Ti_4_AlN_3_ MAX phase reinforced boron based composites is considered good for semiconductor properties because it provides a balance between UV protection and material stability. This range helps in maintaining the structural and electronic integrity of the semiconductor, ensures thermal stability, and optimizes the performance of optoelectronic devices. It minimizes the risks associated with high absorbance, such as material degradation and thermal stress, while allowing for sufficient UV transparency to prevent significant energy loss.^55,56^

#### 4.4.2 UV spectra analysis of Ti_3_AlC_2_ MAX phase reinforced boron based composites

Boron-based Ti_3_AlC_2_ MAX phase composites are often engineered for high strength and durability. Absorbance in UV light can be an indicator of the material's stability and resistance to UV-induced degradation, which is crucial for applications exposed to sunlight or other UV sources over extended periods. UV analysis of Ti_4_AlB_2_C as shown in Fig. 13 indicates that the absorbance rate of fabricated composites varies from 0.008 to 2.4 a.u. with the variation of sintering temperatures from 950 °C to 1325 °C. Fig. 13(a) and (c) indicate high absorbance rates of 2.4 and 1.6 a.u. respectively but these absorbance rates gradually decline with increasing wavelength. Fig. 13(b), (c) and (e) show that the absorbance rates are low which are within the range of 0.008 to 0.65 a.u. A low absorbance rate in the range of 0.008 to 0.65 a.u. (absorbance units) for a boron-based Ti_3_AlC_2_ MAX phase composite in a UV test is highly favorable for semiconductor applications. An absorbance rate in this range indicates that the material allows a significant portion of UV light to pass through it. This transparency is crucial for semiconductor applications where precise control and manipulation of UV light are required, such as in photolithography processes or UV sensors.^57^

Semiconductor devices often rely on the efficient absorption of UV light to generate electrical signals or perform specific functions. A low absorbance rate within this range ensures that the semiconductor material efficiently absorbs UV light, leading to enhanced device performance in terms of sensitivity, response time, and signal-to-noise ratio. The specified range (0.008 to 0.65 a.u.) suggests that the material's absorbance is well-controlled within the desired UV spectral range. This is crucial for semiconductor applications that operate within specific UV wavelengths, ensuring that the material's optical properties align with the requirements of the application. Semiconductor fabrication processes often involve exposure to UV light for various steps such as patterning, doping, or annealing. A boron-based Ti_3_AlC_2_ MAX phase composite with a low absorbance rate within this range is compatible with such processes, minimizing unwanted absorption or interference during manufacturing. Consistent and low absorbance within the specified range indicates good material quality and stability, which are essential for the long-term reliability of semiconductor devices. It suggests that the material's optical properties will remain consistent over time, minimizing performance degradation or drift.^58^

Semiconductors are commonly used in photodetectors, where they convert incident light (UV, in this case) into electrical signals. Low absorbance means more UV light can penetrate the material, increasing the number of photons available for detection. This enhances the photodetection efficiency of the semiconductor device. In conclusion, a boron-based Ti_3_AlC_2_ MAX phase composite with a low absorbance rate falling within the range of 0.008 to 0.65 a.u. in a UV test is highly desirable for semiconductor applications. It offers optical transparency, supports device performance, aligns with specific spectral requirements, ensures process compatibility, and enhances the reliability and stability of semiconductor devices.^51^

#### 4.4.3 Bandgap analysis

The simplest way to investigate bandgap changes caused by different sintering temperatures and different % of MAX phases is UV-vis spectroscopy, which analyses the bandgap (*E*_g_), may be determined using the Tauc plot relationship equation (eqn (5)).^59–63^

**Fig. 14:**
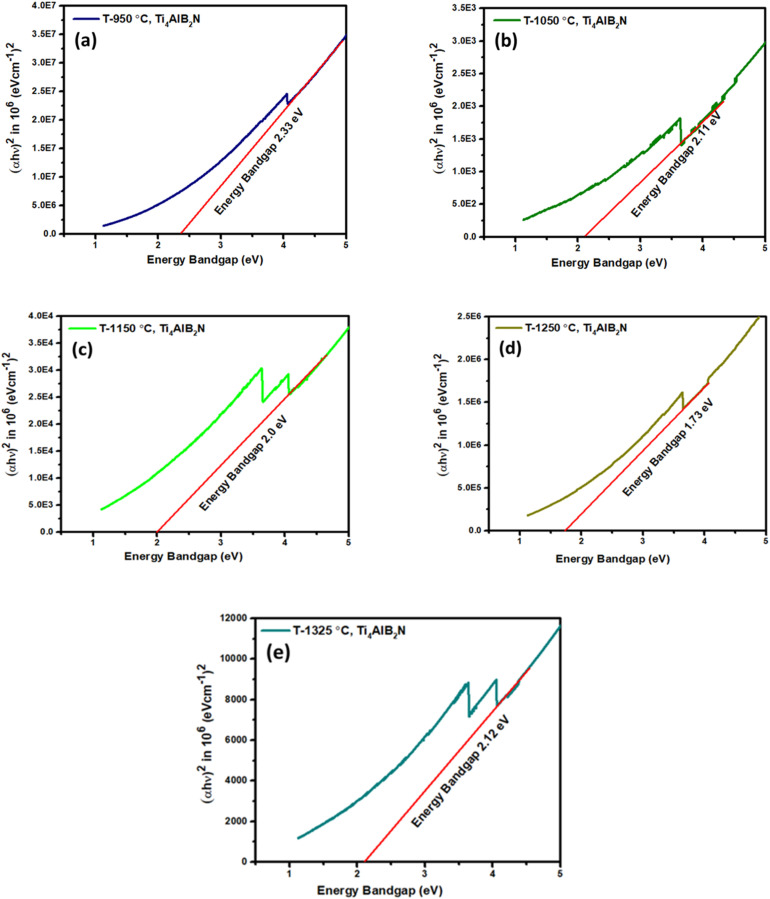
Tauc plot for energy band gaps of Ti_4_AlB_2_N at different sintering temperatures: (a) sample sintered at 950 °C, (b) sample sintered at 1050 °C, (c) sample sintered at 1150 °C, (d) sample sintered at 1250 °C and (e) sample sintered at 1325 °C.

**Fig. 15:**
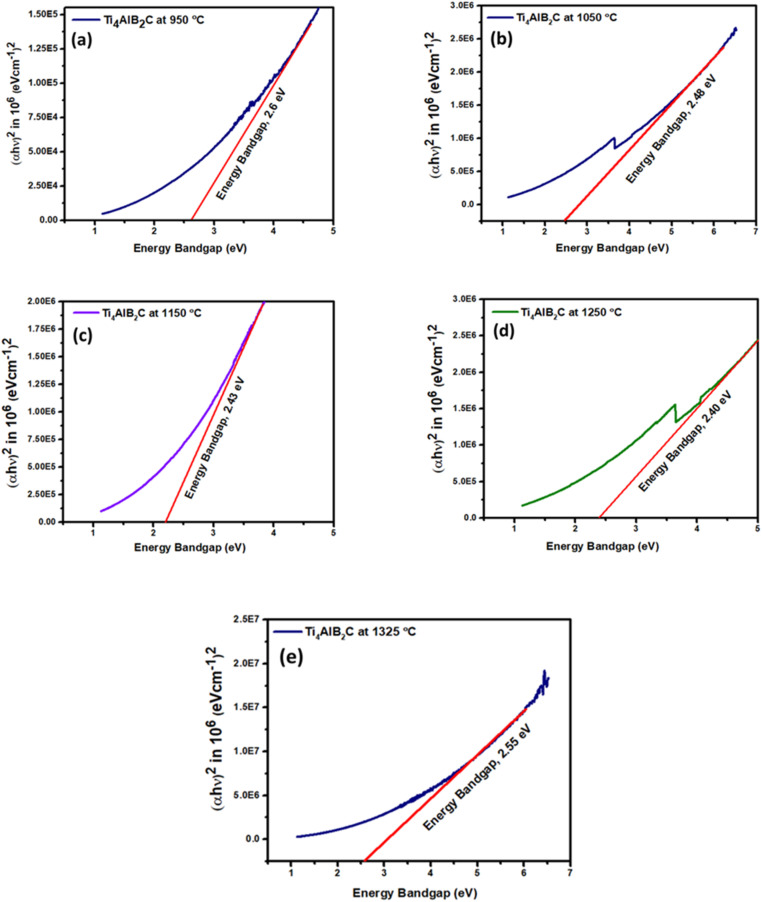
Tauc plot for energy band gaps of Ti_4_AlB_2_C at different sintering temperatures: (a) sample sintered at 950 °C, (b) sample sintered at 1050 °C, (c) sample sintered at 1150 °C, (d) sample sintered at 1250 °C and (e) sample sintered at 1325 °C.

5 (*αhv*)^*n*^ = *A*(*hv* − *E*_g_)

where *A* stands for a characteristic parameter independent of photon energy, *h* is the energy of the incident photon, and *n* is a constant that depends on the transition between the bottom of the conduction band and the top of the valence band. For the direct legal transition, *n* = 2, and for the legal indirect transfer, *n* = 1/2. Plotting (*αhv*)^*n*^*vs.* energy (*hv*) and extending the straight-line segment of the curve towards the *x*-axis yield the optical band gap. The band gap values were estimated using the Tauc plot for Ti_4_AlB_2_N and Ti_4_AlB_2_C samples sintered at different temperatures of 950 °C, 1050 °C, 1150 °C, 1250 °C and 1325 °C as shown in Fig. 14 and 15. Table 5 compares the bandgap, % of the MAX phase and % of the boron of Ti_4_AlB_2_N and Ti_4_AlB_2_C samples sintered at different temperatures of 950 °C, 1050 °C, 1150 °C, 1250 °C and 1325 °C respectively.

Bandgaps of Ti_4_AlB_2_N and Ti_3_AlB_2_C at different sintering temperatures

**Table:** 

Sample compound	Band gap, MAX phase and boron% of prepared samples at different sintering temperatures					
	Sample name	Sample 306C (950 °C)	Sample 206B (1050 °C)	Sample 306A (1150 °C)	Sample 306B (1250 °C)	Sample 206A (1325 °C)
Ti_4_AlB_2_N	Bandgap (eV)	2.33	2.11	2.00	1.73	2.12
	MAX phase (Ti_4_AlN_3_)%	1.4	0.8	0	1.1	2.9

**Table:** 

Sample compound	Band gap, MAX phase and boron% of prepared samples at different sintering temperatures					
	Sample name	Sample 309C (950 °C)	Sample 209B (1050 °C)	Sample 309A (1150 °C)	Sample 309B (1250 °C)	Sample 209A (1325 °C)
Ti_4_AlB_2_C	Bandgap (eV)	2.60	2.48	2.43	2.40	2.55
	MAX phase (Ti_3_AlC_2_)%	0.9	1.7	1.9	2.0	2.0
	Boron%	47.7	85.4	97.5	98	98

##### 4.4.3.1 Bandgap of Ti_4_AlN_3_ MAX phase reinforced boron-based composites (Ti_4_AlB_2_N)

The variation in the bandgap of Ti_4_AlN_3_ MAX phase reinforced boron-based composites (Ti_4_AlB_2_N) with sintering temperature reveals important insights into the material's structural and electronic properties. At a lower sintering temperature, such as 950 °C, the material exhibits a relatively high bandgap of 2.33 eV due to smaller grain sizes and a higher number of defects and grain boundaries. These imperfections create localized states within the bandgap. As the sintering temperature increases to 1050 °C and 1150 °C, the bandgap decreases to 2.11 eV and 2.00 eV, respectively. This reduction is attributed to grain growth and enhanced crystallinity, which diminish the number of defect states. At an optimal sintering temperature of 1250 °C, the material achieves its lowest bandgap of 1.73 eV. This temperature promotes the highest degree of structural order and crystallinity, significantly reducing defects and grain boundaries, and thus minimizing localized electronic states within the bandgap. However, further increasing the temperature to 1325 °C leads to a rise in the bandgap to 2.12 eV. This increase suggests the onset of over-sintering, where additional phases or intergranular reactions occur, reintroducing defects and potentially forming secondary phases, which adversely affect the band structure.

The changes in bandgaps with sintering temperature have substantial implications for semiconductor applications. Materials with a lower bandgap, such as those sintered at 1250 °C, are desirable for electronic and optoelectronic devices due to their efficient charge carrier movement. However, the increase in bandgaps at higher temperatures indicates potential issues with thermal and phase stability, which could limit the material's performance in high-temperature environments.^64,65^ These factors highlight the delicate balance required in the sintering process to achieve desired electronic properties for semiconductor applications. Appropriate control of the sintering temperature is essential to optimize the grain size, reduce defects, and prevent unwanted phase transformations, thereby tailoring the bandgap for specific electronic and optoelectronic uses.^66,67^

##### 4.4.3.2 Bandgap of Ti_3_AlC_2_ MAX phase reinforced boron-based composites (Ti_4_AlB_2_C)

The variation in bandgap values for Ti_3_AlC_2_ MAX phase reinforced boron-based composites (Ti_4_AlB_2_C) with different sintering temperatures can be explained by examining the material's structural and electronic changes during the sintering process. At a lower sintering temperature of 950 °C, the material has a high concentration of defects and smaller grain sizes. Defects such as grain boundaries, vacancies, and interstitials create localized states within the bandgap, leading to a higher bandgap value of 2.60 eV. Additionally, incomplete sintering may result in less optimal crystallinity, contributing to the wider bandgap. With an increase in temperature at 1050 °C, 1150 °C and 1250 °C, the grains start to grow, reducing the number of grain boundaries and defects. This reduction in defect states causes the bandgap to decrease slightly at 2.48 eV, 2.43 eV and 2.40 eV respectively. The balance between reduced defect density and the potential for new defect formation can cause minor fluctuations in the bandgap. The material reaches an optimal sintering state at a temperature of 1250 °C, where the crystallinity is highest and the defect concentration is minimized. Larger grain sizes and fewer grain boundaries contribute to fewer localized electronic states within the bandgap, resulting in the lowest bandgap value of 2.40 eV. This indicates an optimal balance of structural order and electronic properties. Further increasing the temperature at 1325 °C leads to over-sintering, where new defects or secondary phases may form. These new defects or intergranular reactions can introduce localized states within the bandgap, causing an increase in the bandgap of 2.55 eV again. The formation of these secondary phases or defects disrupts the optimal electronic structure achieved at lower temperatures.^68^

The growth of grains and reduction of grain boundaries with increasing temperature initially reduce defect states, lowering the bandgap. However, excessive grain growth or the formation of secondary phases at higher temperatures can reintroduce defects. The concentration of defects such as vacancies, interstitials, and grain boundaries directly affects the bandgap. An optimal sintering temperature minimizes these defects, resulting in the lowest bandgap, while too high or too low sintering temperatures increase defects. At very high temperatures, phase transformations or secondary phase formations can alter the electronic structure, leading to increased bandgap values.^69^

The sintering temperature has a significant impact on the bandgap of Ti_3_AlC_2_ MAX phase reinforced boron-based composites. An optimal sintering temperature around 1250 °C minimizes defects and achieves the lowest bandgap, making the material suitable for high-efficiency semiconductor applications. However, temperatures above this optimal point can introduce new defects or secondary phases, increasing the bandgap and potentially limiting the material's utility in certain applications. Controlling the sintering process is essential to optimize the material's properties for specific electronic and optoelectronic applications.

##### 4.4.3.3 Impact of resultant bandgaps on semiconductor applications

The sintering temperature-dependent bandgap variations as shown in Table 5 for Ti_4_AlN_3_ and Ti_3_AlC_2_ MAX phase reinforced boron-based composites reveal distinct behaviors that affect their suitability for semiconductor applications. For Ti_4_AlN_3_ composites, the bandgap decreases from 2.33 eV at 950 °C to a minimum of 1.73 eV at 1250 °C before increasing to 2.12 eV at 1325 °C. This trend suggests that up to 1250 °C, the material's crystallinity improves, and defects decrease, leading to a lower bandgap. The increase at 1325 °C indicates over-sintering, introducing new defects or secondary phases. In contrast, Ti_3_AlC_2_ composites show a bandgap decrease from 2.60 eV at 950 °C to 2.40 eV at 1250 °C, and then rising to 2.55 eV at 1325 °C. The initial decrease reflects grain growth and reduced defects. The optimal bandgap of 2.40 eV at 1250 °C indicates good sintering conditions, with the increase at 1325 °C again pointing to over-sintering effects.

Materials with lower bandgap values, such as those sintered at 1250 °C, are preferable for applications that require efficient charge carrier movement, such as transistors, solar cells, and light-emitting diodes (LEDs). The minimized defects and optimal electronic properties at this temperature make the material suitable for high-performance applications.^70,71^ The increase in bandgaps at higher temperatures (1325 °C) suggests potential issues with thermal and phase stability. Devices operating at high temperatures may experience changes in electronic properties, affecting performance.^72,73^ The ability to tune the bandgap with sintering temperature allows for customization of the material's optical absorption and emission spectra. This is important for optoelectronic applications, where specific bandgap values are required for different functionalities.^74^

Ti_4_AlN_3_ MAX phase reinforced boron based composites are more suitable for semiconductor applications requiring lower bandgaps, such as optoelectronic devices, due to their minimum bandgap of 1.73 eV at 1250 °C, indicating high efficiency and good crystallinity. On the other hand, Ti_3_AlC_2_ MAX phase reinforced boron based composites, with a higher optimal bandgap of 2.40 eV, are better suited for applications needing higher energy absorption, like UV detectors and high-power electronics. Thus, while Ti_4_AlN_3_ MAX phase reinforced composites offer versatility and efficiency for a broader range of applications, Ti_3_AlC_2_ MAX phase reinforced composites are ideal for specific high-energy applications.

## 5. Conclusions

*In situ* Ti_3_AlC_2_ and Ti_4_AlN_3_ MAX phase reinforced boron-based composites were successfully synthesized using hot pressing and inert sintering. XRD and FESEM confirmed the presence of MAX and secondary phases along with amorphous boron. As sintering temperatures increased, dislocation density decreased and microstrain reduced, enhancing structural integrity. The UV-vis test showed that Ti_4_AlN_3_ composites had absorbance rates from 0.065 a.u. to 0.63 a.u. (1050 °C to 1325 °C), making them suitable for semiconductor applications. Ti_4_AlB_2_C composites showed absorbance rates from 0.008 to 2.4 a.u. The energy bandgap decreased with increasing temperature, from 2.33 eV to 1.73 eV for Ti_4_AlB_2_N and 2.60 eV to 2.40 eV for Ti_4_AlB_2_C. Ti_4_AlN_3_ composites are ideal for optoelectronic devices due to a minimum bandgap of 1.73 eV, while Ti_3_AlC_2_ composites, with a higher bandgap of 2.40 eV, are better for UV detectors and high-power electronics. These composites have versatile applications at room temperature and can serve industries like semiconductors, energy storage, and aerospace. Future research should optimize sintering to refine the bandgap and crystallinity of MAX phase-reinforced boron-based composites, exploring their performance in semiconductor and optoelectronic applications under different conditions.

## Data availability

The data supporting this article are provided as part of the ESI.†

## Author contributions

Md. Shahinoor Alam: conceptualization, analysis, original draft, review and editing. Mohammad Asaduzzaman Chowdhury: supervision, analysis and funding acquisition. Md. Saiful Islam: review and editing. Md. Moynul Islam: investigation and data curation. Md. Abdus Sabur: investigation and software support. Md. Masud Rana: data curation and editing.

## Conflicts of interest

The authors declare that this research paper does not have any financial and personal relationships with other people or organizations.

## Supplementary Material

NA-OLF-D4NA00738G-s001

NA-OLF-D4NA00738G-s002

NA-OLF-D4NA00738G-s003

NA-OLF-D4NA00738G-s004

NA-OLF-D4NA00738G-s005

NA-OLF-D4NA00738G-s006

NA-OLF-D4NA00738G-s007

NA-OLF-D4NA00738G-s008

NA-OLF-D4NA00738G-s009

NA-OLF-D4NA00738G-s010
