# Supplementary material for: Exploring semiconductor potential: novel boron-based Ti3AlC2 and Ti4AlN3 MAX phase composites with tunable band gaps
Source: Nanoscale Adv. 2024 Nov 12;7(1):288–309. doi: 10.1039/d4na00738g (PMC11603385; doi:10.1039/d4na00738g)
Supplement: NA-007-D4NA00738G-s003 [file NA-007-D4NA00738G-s003.pdf]

Supplementary Information (SI) for Nanoscale Advances.
This journal is © The Royal Society of Chemistry 2024

This is the simple example template containing only headers for each report item and the bookmarks. The invisible bookmarks are indicated by text between brackets.
Modify it according to your own needs and standards.

Measurement Conditions: (Bookmark 1)

Dataset Name 209(A)- Ti4AlB2C Optimized_2
File name E:\XRD data files\New Analysis-Dr. Moynul-BAUET\1st slot-XRD (1325 C)-04\209(A)- Ti4AlB2C-1325C\209(A)- Ti4AlB2C Optimized_2.xrdml
Comment Configuration=Flat Sample Stage, Owner=pc, Creation date=11/22/2023 7:32:59 PM
Goniometer=Theta/Theta; Minimum step size 2Theta:0.0001; Minimum step size Omega:0.0001
Sample stage=Stage for flat samples/holders
Diffractometer system=EMPYREAN
Measurement program=C:\PANalytical\Data Collector\Programs\2B Ti3AlB2 Optimized.xrdmp, Created identifier={1511DE26-B7FE-4D9E-AD02-FB0BF5A869E9}, Created by=pc, Id=WL:DESKTOP-BC0RBBD:pc, Creation date=1/18/2024 6:31:02 PM
PHD Lower Level = 7.89 (keV), PHD Upper Level = 11.27 (keV)

Measurement Start Date/Time	1/20/2024 5:14:26 PM
Operator	pc
Raw Data Origin	XRD measurement (*.XRDML)
Scan Axis	Gonio
Start Position [°2θ]	10.0096
End Position [°2θ]	89.9986
Step Size [°2θ]	0.0130
Scan Step Time [s]	18.8700
Scan Type	Continuous
PSD Mode	Scanning
PSD Length [°2θ]	3.35
Offset [°2θ]	0.0000
Divergence Slit Type	Fixed
Divergence Slit Size [°]	0.7197
Specimen Length [mm]	10.00
Measurement Temperature [°C]	25.00
Anode Material	Cu
Intended Wavelength Type	K-α1
K-α1 [Å]	1.54060
K-α2 [Å]	1.54443
K-β1 [Å]	1.39225
K-β2 [Å]	1.38113
K-β3 [Å]	1.39261
K-A2 / K-A1 Ratio	0.50000
K-Alpha2 Line Shift	0.00000
K Absorption Edge	1.37868
Generator Settings	40 mA, 45 kV
Diffractometer Type	0000000011286122
Diffractometer Number	0
Goniometer Radius [mm]	240.00

Dist. Focus-Diverg. Slit [mm]	60.50
Incident Beam Monochromator	No
Spinning	No

Main Graphics, Analyze View: (Bookmark 2)

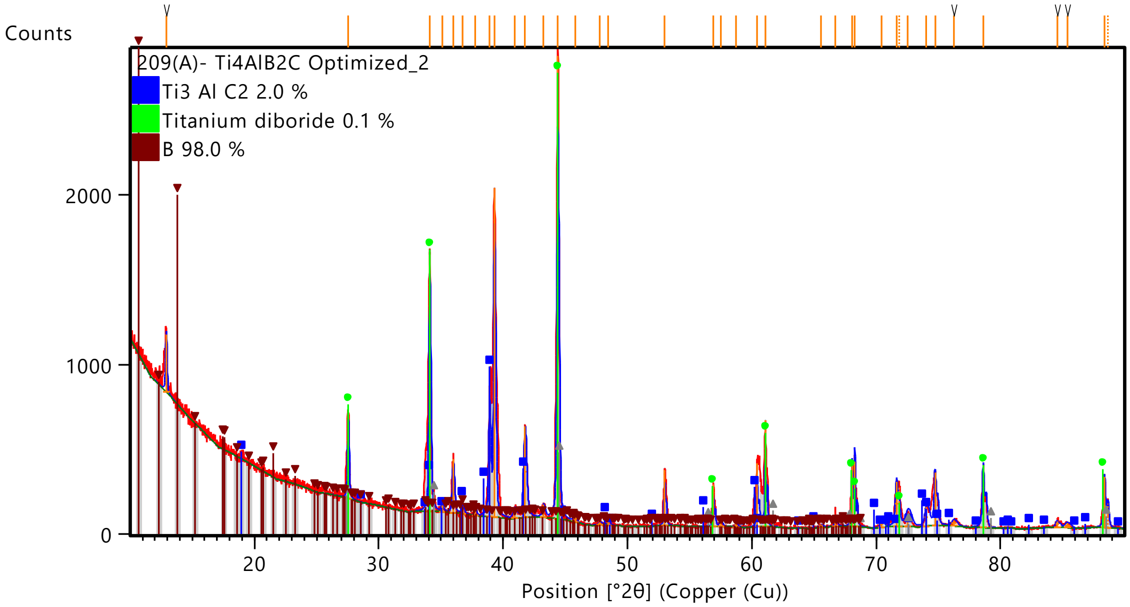

Peak List: (Bookmark 3)

Pos. [°2θ]	Height [cts]	FWHM Left [°2θ]	d-spacing [Å]	Rel. Int. [%]
12.9137	339.66	0.1279	6.84984	12.45
27.5417	507.14	0.1279	3.23601	18.59
34.0956	1517.17	0.1535	2.62749	55.61
35.1021	58.12	0.1535	2.55442	2.13
35.9884	317.70	0.1791	2.49351	11.64
36.7305	46.21	0.2558	2.44482	1.69
37.7534	47.63	0.1535	2.38090	1.75
38.9034	766.90	0.1535	2.31313	28.11
39.2999	1938.32	0.1535	2.29070	71.04
40.9479	56.87	0.3070	2.20223	2.08
41.7548	537.99	0.2558	2.16152	19.72
43.2563	75.46	0.2047	2.08990	2.77
44.3941	2728.35	0.1535	2.03894	100.00
45.7798	34.35	0.2558	1.98040	1.26
47.7950	18.57	0.3070	1.90150	0.68
48.4563	34.69	0.2047	1.87708	1.27
52.9975	329.33	0.1279	1.72644	12.07
56.9116	245.27	0.1023	1.61664	8.99
57.4775	41.87	0.2047	1.60206	1.53
58.7583	16.41	0.3070	1.57016	0.60
60.4259	375.75	0.1279	1.53075	13.77

61.0848	625.32	0.1023	1.51581	22.92
65.5774	13.92	0.3070	1.42242	0.51
66.7001	69.74	0.1535	1.40117	2.56
68.0633	381.01	0.1248	1.37639	13.96
68.2879	380.95	0.1023	1.37241	13.96
70.4243	41.63	0.2558	1.33592	1.53
71.6423	260.35	0.2184	1.31617	9.54
71.8780	232.74	0.1560	1.31243	8.53
72.5584	75.15	0.4992	1.30180	2.75
74.0464	110.84	0.3120	1.27927	4.06
74.7394	311.74	0.1872	1.26912	11.43
76.2575	32.17	0.3744	1.24759	1.18
78.6092	364.25	0.1872	1.21605	13.35
84.5438	36.89	0.2496	1.14517	1.35
85.3526	27.82	0.3744	1.13638	1.02
88.3648	301.78	0.1872	1.10525	11.06
88.6568	141.59	0.1560	1.10236	5.19

Pattern List: (Bookmark 4)

Visible	Ref.Code	Score	Compound Name	Displ.[°2θ]	Scale Fac.	Chem. Formula
*	96-722-1325	24	Ti3 Al C2	0.000	0.307	Ti6.00 C4.00 Al2.00
*	96-200-2801	62	Titanium diboride	0.000	0.915	Ti1.00 B2.00
*	96-153-2806	6	1532805	0.000	0.137	Al0.91 B2.00
*	96-151-1439	0	1511438	0.000	0.862	B189.88

Document History: (Bookmark 5)

ESD calculated from counts:
- Modification time = "2/14/2024 9:51:47 AM"
- Modification editor = "pc"

Insert Measurement:
- File name = "209(A)- Ti4AlB2C Optimized_2.xrdml"
- Modification time = "2/14/2024 9:51:47 AM"
- Modification editor = "pc"

Default properties:
- Measurement step axis = "None"
- Internal wavelengths used from anode material: Copper (Cu)
- Original K-Alpha1 wavelength = "1.54060"
- Used K-Alpha1 wavelength = "1.54060"
- Original K-Alpha2 wavelength = "1.54443"
- Used K-Alpha2 wavelength = "1.54443"
- Original K-Beta wavelength = "1.39225"
- Used K-Beta wavelength = "1.39225"

- Irradiated length = "10.00000"
- Spinner used = "No"
- KBeta filter material = "Ni"
- KBeta filter thickness = "0.02000"
- Receiving slit size = "0.10000"
- Step axis value = "0.00000"
- Offset = "0.00000"
- Sample length = "10.00000"
- Modification time = "2/14/2024 9:51:47 AM"
- Modification editor = "pc"

Interpolate Step Size:
- Initial Scan Range = 7.00657 - 90.00310
- Initial Step Size = 0.01313
- Derived Step Size = 0.01300
- Use Derived Step Size = "Yes"
- Parameterset name = "Default"
- PANalytical factory default
- Modification time = "2/14/2024 9:51:47 AM"
- Modification editor = "pc"

Clip Range:
- Old/New start = "7.0066/10.0000"
- Old/New end = "89.9986/89.9986"
- Modification time = "2/14/2024 9:53:08 AM"
- Modification editor = "pc"

Determine Background:
- Add to net scan = "Nothing"
- User defined intensity = "-5"
- Correction method = "Automatic"
- Bending factor = "1"
- Minimum significance = "0.7"
- Minimum tip width = "0"
- Maximum tip width = "1"
- Peak base width = "2"
- Use smoothed input data = "Yes"
- Granularity = "10"
- Search window = "5"
- Spline type = "Linear"
- Parameterset name = "Untitled"
- Parameterset modification time = "2/9/2024 8:08:11 PM"
- Parameterset modification editor = "pc"
- Modification time = "2/14/2024 9:53:18 AM"
- Modification editor = "pc"

Search Peaks:
- Minimum significance = "2"
- Minimum tip width = "0.1"
- Maximum tip width = "1"
- Peak base width = "2"

- Method = "Minimum 2nd derivative"
- Parameterset name = "Untitled"
- Parameterset modification time = "2/13/2024 3:42:23 PM"
- Parameterset modification editor = "pc"
- Modification time = "2/14/2024 9:53:30 AM"
- Modification editor = "pc"

Search & Match:
- Allow pattern shift = "No"
- Auto residue = "Yes"
- Data source = "Profile and peak list"
- Demote unmatched strong = "Yes"
- Multi phase = "Yes"
- Restriction set = "Untitled"
- Restriction = "Restriction set"
- Subset name = ""
- Match intensity = "Yes"
- Two theta shift = "0"
- Identify = "No"
- Max. no. of accepted patterns = "5"
- Minimum score = "50"
- Min. new lines / total lines = "60"
- Search depth = "10"
- Minimum new lines = "5"
- Minimum scale factor = "0.1"
- Intensity threshold = "0"
- Use line clustering = "Yes"
- Line cluster range = "1.5"
- Search sensitivity = "1.8"
- Use adaptive smoothing = "Yes"
- Smoothing range = "1.5"
- Threshold factor = "3"
- Match Threshold = "0"
- N * Esds = "-1"
- Raw Weight = "-1"
- Peak Shape = "-1"
- Accepted Shape = "-1"
- Peak Power = "-1"
- New Peak Power = "-1"
- Intensity Power = "-1"
- N Peaks Power = "-1"
- Parameterset name = "Untitled"
- Parameterset modification time = "2/14/2024 9:55:11 AM"
- Parameterset modification editor = "pc"
- Modification time = "2/14/2024 9:55:14 AM"
- Modification editor = "pc"

Convert Ref. Pattern to Phase:
- Modification time = "2/14/2024 9:57:34 AM"
- Modification editor = "pc"

Edit 1532805 Title:
- Old Value = "1532805"
- Modification time = "2/14/2024 9:58:08 AM"
- Modification editor = "pc"

Edit 1511438 Title:
- Old Value = "1511438"
- Modification time = "2/14/2024 9:58:15 AM"
- Modification editor = "pc"

Edit Ti3 Al C2 Asymmetry Type:
- Old Value = "No Asymmetry Function"
- Modification time = "2/14/2024 9:59:58 AM"
- Modification editor = "pc"

Edit Titanium diboride Asymmetry Type:
- Old Value = "No Asymmetry Function"
- Modification time = "2/14/2024 10:00:03 AM"
- Modification editor = "pc"

Edit AlB2 Asymmetry Type:
- Old Value = "No Asymmetry Function"
- Modification time = "2/14/2024 10:00:07 AM"
- Modification editor = "pc"

Edit B Asymmetry Type:
- Old Value = "No Asymmetry Function"
- Modification time = "2/14/2024 10:00:13 AM"
- Modification editor = "pc"

Edit Solver Tolerance:
- Old Value = "0.001"
- Modification time = "2/14/2024 10:00:24 AM"
- Modification editor = "pc"

Delete Selected Phase:
- Modification time = "2/14/2024 10:00:40 AM"
- Modification editor = "pc"

XRD Measurement Information: (Bookmark 6)

More items… (Bookmark 7)

More items… (Bookmark 8)

More items… (Bookmark 9)

More items… (Bookmark 10)

More items… (Bookmark 11)

More items… (Bookmark 12)

More items… (Bookmark 13)

More items… (Bookmark 14)

More items… (Bookmark 15)
