# Supplementary material for: Exploring semiconductor potential: novel boron-based Ti3AlC2 and Ti4AlN3 MAX phase composites with tunable band gaps
Source: Nanoscale Adv. 2024 Nov 12;7(1):288–309. doi: 10.1039/d4na00738g (PMC11603385; doi:10.1039/d4na00738g)
Supplement: NA-007-D4NA00738G-s004 [file NA-007-D4NA00738G-s004.pdf]

Supplementary Information (SI) for Nanoscale Advances.
This journal is © The Royal Society of Chemistry 2024

This is the simple example template containing only headers for each report item and the bookmarks. The invisible bookmarks are indicated by text between brackets.
Modify it according to your own needs and standards.

Measurement Conditions: (Bookmark 1)

Dataset Name	206(B)- Ti4AlB2N Optimized_3
File name	E:\XRD data files\New Analysis-Dr. Moynul-BAUET\2nd slot-XRD (1050 C)-06\206(B)- Ti4AlB2N-1050C\206(B)- Ti4AlB2N Optimized_3.xrdml
Comment	Configuration=Flat Sample Stage, Owner=pc, Creation date=11/22/2023 7:32:59 PM<br>Goniometer=Theta/Theta; Minimum step size 2Theta:0.0001; Minimum step size Omega:0.0001<br>Sample stage=Stage for flat samples/holders<br>Diffractometer system=EMPYREAN<br>Measurement program=C:\PANalytical\Data Collector\Programs\2B Ti3AlB2 Optimized.xrdmp, Created identifier={1511DE26-B7FE-4D9E-AD02-FB0BF5A869E9}, Created by=pc, Id=WL:DESKTOP-BC0RBBD:pc, Creation date=1/18/2024 6:31:02 PM, Modified identifier={706E23D6-EF3A-4959-B077-79A506FC4E68}, Modified by=pc, Id=WL:DESKTOP-BC0RBBD:pc, Modification date=2/7/2024 9:05:13 PM<br>PHD Lower Level = 4.02 (keV), PHD Upper Level = 16.10 (keV)
Measurement Start Date/Time	2/9/2024 8:16:56 PM
Operator	pc
Raw Data Origin	XRD measurement (*.XRDML)
Scan Axis	Gonio
Start Position [°2θ]	20.0348
End Position [°2θ]	89.9288
Step Size [°2θ]	0.0660
Scan Step Time [s]	12.7500
Scan Type	Continuous
PSD Mode	Scanning
PSD Length [°2θ]	3.35
Offset [°2θ]	0.0000
Divergence Slit Type	Fixed
Divergence Slit Size [°]	0.7197
Specimen Length [mm]	10.00
Measurement Temperature [°C]	25.00
Anode Material	Cu
Intended Wavelength Type	K-α1
K-α1 [Å]	1.54060
K-α2 [Å]	1.54443
K-β1 [Å]	1.39225
K-β2 [Å]	1.38113
K-β3 [Å]	1.39261
K-A2 / K-A1 Ratio	0.50000
K-Alpha2 Line Shift	0.00000
K Absorption Edge	1.37868
Generator Settings	40 mA, 45 kV
Diffractometer Type	0000000011286122

Diffractometer Number	0
Goniometer Radius [mm]	240.00
Dist. Focus-Diverg. Slit [mm]	60.50
Incident Beam Monochromator	No
Spinning	No

Main Graphics, Analyze View: (Bookmark 2)

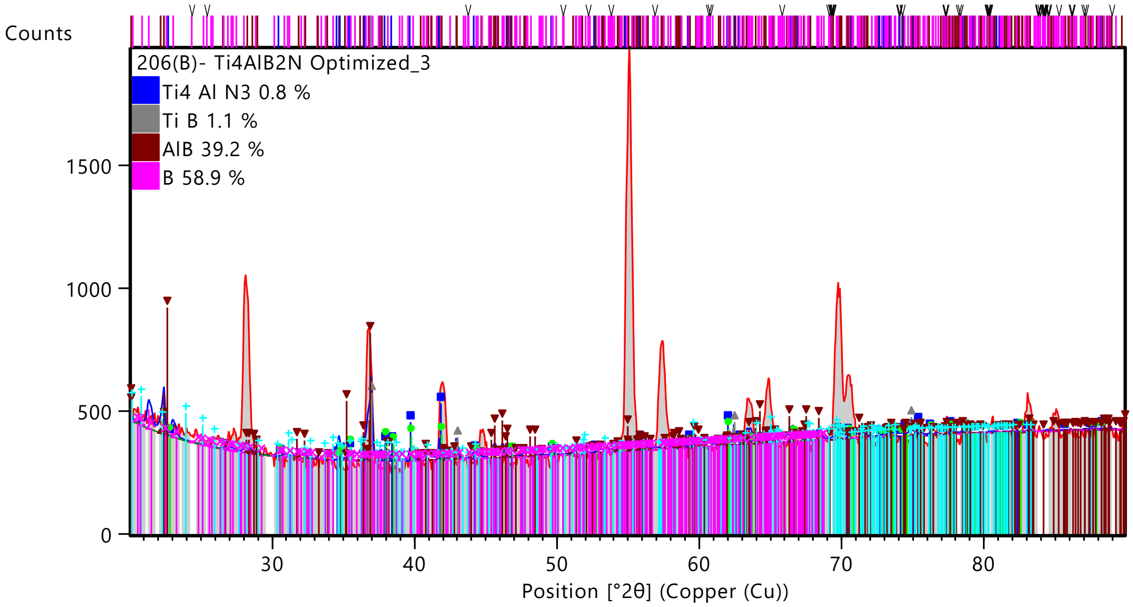

Peak List: (Bookmark 3)

Pos. [°2θ]	Height [cts]	FWHM Left [°2θ]	d-spacing [Å]	Rel. Int. [%]
20.1012	20.31	0.3618	4.41388	8.72
20.1948	3.33	0.3025	4.39362	1.43
20.1948	2.58	0.3025	4.39362	1.11
20.8512	11.32	0.3025	4.25676	4.86
21.3377	79.52	0.3025	4.16081	34.16
22.1865	19.81	0.3618	4.00350	8.51
22.4195	165.87	0.3618	3.96241	71.24
22.5755	6.16	0.3025	3.93539	2.65
22.7118	0.35	0.1000	3.91208	0.15
23.0274	23.37	0.3025	3.85917	10.04
23.0274	25.64	0.3025	3.85917	11.01
24.3296	2.16	0.3025	3.65548	0.93
25.1608	1.94	0.3025	3.53658	0.83
25.4245	1.08	0.3025	3.50048	0.46
25.6971	1.09	0.3025	3.46397	0.47
25.8352	0.25	0.3025	3.44576	0.11
26.5444	0.02	0.3618	3.35529	0.01
26.6225	5.12	0.3025	3.34562	2.20
26.8730	0.28	0.3025	3.31500	0.12

27.1321	4.92	0.3025	3.28393	2.11
27.1321	4.06	0.3025	3.28393	1.74
27.2635	0.54	0.3025	3.26841	0.23
27.2635	0.17	0.3025	3.26841	0.07
27.5138	0.37	0.3025	3.23923	0.16
28.0137	2.07	0.3025	3.18256	0.89
28.0137	1.20	0.3025	3.18256	0.51
28.2394	16.49	0.3618	3.15763	7.08
28.2527	2.17	0.3025	3.15617	0.93
28.2577	17.20	0.3618	3.15563	7.39
28.6294	17.27	0.3618	3.11550	7.42
28.8655	1.89	0.3025	3.09055	0.81
28.9788	10.17	0.3618	3.07872	4.37
30.1617	1.66	0.3025	2.96061	0.71
30.1617	1.23	0.3025	2.96061	0.53
30.4692	0.94	0.1000	2.93144	0.40
30.8427	6.59	0.3025	2.89678	2.83
31.0715	2.63	0.3025	2.87597	1.13
31.1877	0.17	0.3025	2.86552	0.07
31.1877	0.97	0.3025	2.86552	0.41
31.8334	23.62	0.3618	2.80886	10.15
31.8537	1.70	0.3025	2.80711	0.73
31.8537	1.68	0.3025	2.80711	0.72
32.0667	0.38	0.3025	2.78895	0.17
32.0667	1.09	0.3025	2.78895	0.47
32.2877	0.01	0.3025	2.77036	0.00
32.3335	22.05	0.3618	2.76654	9.47
33.1407	2.51	0.3025	2.70098	1.08
33.2340	0.20	0.3618	2.69361	0.08
33.4645	9.79	0.3025	2.67558	4.21
33.4645	12.69	0.3025	2.67558	5.45
33.7812	3.74	0.3025	2.65122	1.61
33.7812	4.55	0.3025	2.65122	1.96
33.8749	0.14	0.3618	2.64410	0.06
34.2916	4.94	0.3025	2.61292	2.12
34.5274	3.86	0.1000	2.59561	1.66
34.6928	78.98	0.3618	2.58362	33.92
34.7116	5.41	0.3025	2.58226	2.33
34.7456	8.86	0.1000	2.57981	3.81
35.1118	0.42	0.3618	2.55374	0.18
35.3185	0.93	0.3025	2.53926	0.40
35.3931	14.34	0.1000	2.53408	6.16
35.5132	14.28	0.3025	2.52579	6.13
35.8182	6.22	0.3025	2.50497	2.67
35.8182	6.36	0.3025	2.50497	2.73
36.2060	6.55	0.3618	2.47903	2.81
36.3866	35.94	0.3618	2.46714	15.44
36.4091	1.20	0.3025	2.46566	0.52
36.4091	1.00	0.3025	2.46566	0.43
36.4502	3.87	0.1000	2.46298	1.66

36.4979	6.63	0.3025	2.45987	2.85
36.5988	24.75	0.3025	2.45332	10.63
36.7958	3.29	0.3025	2.44063	1.41
36.7958	3.53	0.3025	2.44063	1.51
36.8322	162.35	0.3618	2.43831	69.73
36.8960	13.50	0.3025	2.43423	5.80
36.9526	232.82	0.1000	2.43064	100.00
37.0876	0.35	0.3025	2.42210	0.15
37.4724	3.54	0.3025	2.39811	1.52
37.5589	0.72	0.3025	2.39278	0.31
37.5589	0.27	0.3025	2.39278	0.12
37.5941	16.51	0.3618	2.39062	7.09
37.6574	3.19	0.3025	2.38675	1.37
37.6574	4.46	0.3025	2.38675	1.92
37.7391	15.94	0.3618	2.38177	6.85
37.8873	23.94	0.1000	2.37279	10.28
38.1347	0.42	0.3025	2.35797	0.18
38.1347	0.63	0.3025	2.35797	0.27
38.3733	24.05	0.1000	2.34385	10.33
38.9750	1.49	0.3025	2.30904	0.64
38.9750	1.53	0.3025	2.30904	0.66
39.1580	0.02	0.3025	2.29867	0.01
39.5256	1.56	0.3025	2.27813	0.67
39.5256	2.15	0.3025	2.27813	0.92
39.6046	0.04	0.3025	2.27378	0.02
39.6700	51.82	0.1000	2.27017	22.26
39.8866	6.09	0.3025	2.25834	2.62
40.4131	0.03	0.3618	2.23013	0.01
40.4532	0.32	0.3618	2.22801	0.14
40.5968	4.41	0.3025	2.22046	1.89
40.6006	2.28	0.3025	2.22026	0.98
40.6929	0.86	0.3025	2.21544	0.37
40.6929	0.49	0.3025	2.21544	0.21
40.8732	1.80	0.3025	2.20608	0.77
40.8732	0.84	0.3025	2.20608	0.36
40.9436	11.79	0.3618	2.20245	5.06
41.1405	5.02	0.3025	2.19236	2.15
41.1405	4.32	0.3025	2.19236	1.85
41.2264	0.06	0.3618	2.18799	0.03
41.5731	1.95	0.3025	2.17054	0.84
41.6550	6.92	0.3618	2.16646	2.97
41.7623	75.39	0.1000	2.16115	32.38
41.8405	6.33	0.3025	2.15729	2.72
41.9210	2.76	0.3618	2.15333	1.19
42.5238	3.77	0.3025	2.12419	1.62
42.5274	1.64	0.3025	2.12402	0.71
42.5274	1.33	0.3025	2.12402	0.57
42.9450	76.29	0.1000	2.10433	32.77
42.9737	7.96	0.3618	2.10299	3.42
43.4612	0.31	0.3025	2.08052	0.13

43.4612	0.23	0.3025	2.08052	0.10
43.5521	0.39	0.3025	2.07639	0.17
43.7228	0.48	0.3025	2.06868	0.21
43.7228	0.86	0.3025	2.06868	0.37
43.8098	0.02	0.3025	2.06477	0.01
43.8098	0.08	0.3025	2.06477	0.03
43.8098	0.01	0.3025	2.06477	0.00
44.1301	9.12	0.1000	2.05053	3.92
44.1312	7.62	0.3618	2.05048	3.27
44.3109	9.01	0.3025	2.04258	3.87
44.3109	5.20	0.3025	2.04258	2.23
44.3109	4.64	0.3025	2.04258	1.99
44.4722	0.62	0.3025	2.03554	0.26
44.4722	0.75	0.3025	2.03554	0.32
44.7019	0.02	0.3618	2.02562	0.01
44.7256	0.11	0.3025	2.02460	0.05
44.7256	0.24	0.3025	2.02460	0.10
45.2187	0.37	0.3025	2.00366	0.16
45.2187	0.55	0.3025	2.00366	0.24
45.2929	1.86	0.3025	2.00055	0.80
45.2929	0.98	0.3025	2.00055	0.42
45.3535	1.89	0.3618	1.99802	0.81
45.3775	0.32	0.3025	1.99702	0.14
45.5536	23.31	0.3618	1.98970	10.01
45.6898	40.83	0.3618	1.98409	17.54
45.7881	0.26	0.3025	1.98006	0.11
45.7881	0.21	0.3025	1.98006	0.09
45.7881	0.60	0.3025	1.98006	0.26
45.8497	1.32	0.3618	1.97754	0.56
46.1822	0.21	0.3025	1.96407	0.09
46.1831	47.28	0.3618	1.96404	20.31
46.2587	6.96	0.3025	1.96101	2.99
46.4730	0.77	0.1000	1.95246	0.33
46.5386	17.87	0.3618	1.94986	7.68
46.6004	28.13	0.3618	1.94742	12.08
46.6735	0.44	0.3025	1.94454	0.19
46.6735	0.36	0.3025	1.94454	0.15
46.7430	4.71	0.1000	1.94181	2.02
46.8187	3.13	0.3618	1.93885	1.34
47.0619	0.85	0.3025	1.92939	0.37
47.1372	1.31	0.3025	1.92648	0.56
47.1473	0.34	0.3025	1.92610	0.14
47.1473	0.55	0.3025	1.92610	0.24
47.3896	0.00	0.3025	1.91681	0.00
47.3896	0.00	0.3025	1.91681	0.00
47.6886	2.51	0.3618	1.90549	1.08
47.8325	26.29	0.3618	1.90009	11.29
47.8614	1.42	0.3025	1.89901	0.61
47.8614	2.00	0.3025	1.89901	0.86
47.9292	0.69	0.3025	1.89649	0.30

47.9292	1.15	0.3025	1.89649	0.49

Pattern List: (Bookmark 4)

Visible	Ref.Code	Score	Compound Name	Displ.[°2θ]	Scale Fac.	Chem. Formula
*	96-152-6339	9	Ti4 Al N3	0.000	0.115	Al2.00 N6.00 Ti8.00
*	96-154-0933	7	Al2 Ti3 N2	0.000	0.102	N4.00 Ti6.00 Al4.00
*	96-151-1333	8	Ti B	0.000	0.140	Ti4.00 B4.00
*	96-156-5387	6	1565386	0.000	0.262	Al5.37 B100.00
*	96-901-1110	10	Boron	0.000	0.171	B315.00
*	96-151-1439	No Matching Lines	1511438	0.000	0.000	B189.88

Document History: (Bookmark 5)

ESD calculated from counts:
- Modification time = "2/14/2024 10:15:50 AM"
- Modification editor = "pc"

Insert Measurement:
- File name = "206(B)- Ti4AlB2N Optimized_3.xrdml"
- Modification time = "2/14/2024 10:15:50 AM"
- Modification editor = "pc"

Default properties:
- Measurement step axis = "None"
- Internal wavelengths used from anode material: Copper (Cu)
- Original K-Alpha1 wavelength = "1.54060"
- Used K-Alpha1 wavelength = "1.54060"
- Original K-Alpha2 wavelength = "1.54443"
- Used K-Alpha2 wavelength = "1.54443"
- Original K-Beta wavelength = "1.39225"
- Used K-Beta wavelength = "1.39225"
- Irradiated length = "10.00000"
- Spinner used = "No"
- KBeta filter material = "Ni"
- KBeta filter thickness = "0.02000"
- Receiving slit size = "0.10000"
- Step axis value = "0.00000"
- Offset = "0.00000"

- Sample length = "10.00000"
- Modification time = "2/14/2024 10:15:50 AM"
- Modification editor = "pc"

Interpolate Step Size:
- Initial Scan Range = 7.03283 - 89.95060
- Initial Step Size = 0.06565
- Derived Step Size = 0.06600
- Use Derived Step Size = "Yes"
- Parameterset name = "Default"
- PANalytical factory default
- Modification time = "2/14/2024 10:15:50 AM"
- Modification editor = "pc"

Clip Range:
- Old/New start = "7.0328/20.0000"
- Old/New end = "89.9288/89.9288"
- Modification time = "2/14/2024 10:16:42 AM"
- Modification editor = "pc"

Determine Background:
- Add to net scan = "Nothing"
- User defined intensity = "-5"
- Correction method = "Automatic"
- Bending factor = "1"
- Minimum significance = "0.7"
- Minimum tip width = "0"
- Maximum tip width = "1"
- Peak base width = "2"
- Use smoothed input data = "Yes"
- Granularity = "10"
- Search window = "5"
- Spline type = "Linear"
- Parameterset name = "Untitled"
- Parameterset modification time = "2/9/2024 8:08:11 PM"
- Parameterset modification editor = "pc"
- Modification time = "2/14/2024 10:16:52 AM"
- Modification editor = "pc"

Search Peaks:
- Minimum significance = "2"
- Minimum tip width = "0.1"
- Maximum tip width = "1"
- Peak base width = "2"
- Method = "Minimum 2nd derivative"
- Parameterset name = "Untitled"
- Parameterset modification time = "2/13/2024 3:42:23 PM"
- Parameterset modification editor = "pc"
- Modification time = "2/14/2024 10:17:04 AM"
- Modification editor = "pc"

Search & Match:
- Allow pattern shift = "No"
- Auto residue = "Yes"
- Data source = "Profile and peak list"
- Demote unmatched strong = "Yes"
- Multi phase = "Yes"
- Restriction set = "Untitled"
- Restriction = "Restriction set"
- Subset name = ""
- Match intensity = "Yes"
- Two theta shift = "0"
- Identify = "No"
- Max. no. of accepted patterns = "5"
- Minimum score = "50"
- Min. new lines / total lines = "60"
- Search depth = "10"
- Minimum new lines = "5"
- Minimum scale factor = "0.1"
- Intensity threshold = "0"
- Use line clustering = "Yes"
- Line cluster range = "1.5"
- Search sensitivity = "1.8"
- Use adaptive smoothing = "Yes"
- Smoothing range = "1.5"
- Threshold factor = "3"
- Match Threshold = "0"
- N * Esds = "-1"
- Raw Weight = "-1"
- Peak Shape = "-1"
- Accepted Shape = "-1"
- Peak Power = "-1"
- New Peak Power = "-1"
- Intensity Power = "-1"
- N Peaks Power = "-1"
- Parameterset name = "Untitled"
- Parameterset modification time = "2/14/2024 10:20:11 AM"
- Parameterset modification editor = "pc"
- Modification time = "2/14/2024 10:20:16 AM"
- Modification editor = "pc"

Convert Ref. Pattern to Phase:
- Modification time = "2/14/2024 10:23:25 AM"
- Modification editor = "pc"

Edit 1565386 Title:
- Old Value = "1565386"
- Modification time = "2/14/2024 10:24:02 AM"
- Modification editor = "pc"

Edit 1511438 Title:
- Old Value = "1511438"

- Modification time = "2/14/2024 10:24:11 AM"
- Modification editor = "pc"

Edit Solver Tolerance:
- Old Value = "0.001"
- Modification time = "2/14/2024 10:24:25 AM"
- Modification editor = "pc"

Edit Ti4 Al N3 Asymmetry Type:
- Old Value = "No Asymmetry Function"
- Modification time = "2/14/2024 10:24:50 AM"
- Modification editor = "pc"

Edit Al2 Ti3 N2 Asymmetry Type:
- Old Value = "No Asymmetry Function"
- Modification time = "2/14/2024 10:24:54 AM"
- Modification editor = "pc"

Edit Ti B Asymmetry Type:
- Old Value = "No Asymmetry Function"
- Modification time = "2/14/2024 10:24:59 AM"
- Modification editor = "pc"

Edit AlB Asymmetry Type:
- Old Value = "No Asymmetry Function"
- Modification time = "2/14/2024 10:25:03 AM"
- Modification editor = "pc"

Edit Boron Asymmetry Type:
- Old Value = "No Asymmetry Function"
- Modification time = "2/14/2024 10:25:08 AM"
- Modification editor = "pc"

Edit B Asymmetry Type:
- Old Value = "No Asymmetry Function"
- Modification time = "2/14/2024 10:25:12 AM"
- Modification editor = "pc"

Profile/Rietveld fitting:
- Angular range Gonio = 20.035 - 89.929
- Step No. 1
- Title = "Scale Factor"
- Switch off after usage = "No"
- Step No. 2
- Title = "Flat Background"
- Switch off after usage = "No"
- Step No. 3
- Title = "More background"
- Switch off after usage = "No"
- No. additional parameters = "2"
- Use 1/X background too = "Yes"

- Step No. 4
- Title = "Specimen Displacement"
- Switch off after usage = "No"
- Step No. 5
- Title = "Lattice Parameters"
- Switch off after usage = "No"
- Minimum weight % = "5"
- Step No. 6
- Title = "Caglioti W"
- Switch off after usage = "No"
- Minimum weight % = "5"
- No. of refined parameters = 20
- Chi Square = 4.50962221229194
- Rp = 0.14302
- Rwp = 0.22448
- Rexp = 0.04978
- BCLM Exit Code = Refinement iterations exceeded
- Modification time = "2/14/2024 10:26:14 AM"
- Modification editor = "pc"

Delete Selected Phase:
- Modification time = "2/14/2024 10:26:21 AM"
- Modification editor = "pc"

Delete Selected Phase:
- Modification time = "2/14/2024 10:26:27 AM"
- Modification editor = "pc"

XRD Measurement Information: (Bookmark 6)

More items… (Bookmark 7)

More items… (Bookmark 8)

More items… (Bookmark 9)

More items… (Bookmark 10)

More items… (Bookmark 11)

More items… (Bookmark 12)

More items… (Bookmark 13)

More items… (Bookmark 14)

More items… (Bookmark 15)
