# Supplementary material for: Exploring semiconductor potential: novel boron-based Ti3AlC2 and Ti4AlN3 MAX phase composites with tunable band gaps
Source: Nanoscale Adv. 2024 Nov 12;7(1):288–309. doi: 10.1039/d4na00738g (PMC11603385; doi:10.1039/d4na00738g)
Supplement: NA-007-D4NA00738G-s005 [file NA-007-D4NA00738G-s005.pdf]

Supplementary Information (SI) for Nanoscale Advances.
This journal is © The Royal Society of Chemistry 2024

This is the simple example template containing only headers for each report item and the bookmarks. The invisible bookmarks are indicated by text between brackets.
Modify it according to your own needs and standards.

Measurement Conditions: (Bookmark 1)

Dataset Name 306(A)- Ti4AlB2N optimised
File name E:\XRD data files\New Analysis-Dr. Moynul-BAUET\3rd phase-XRD (1150 C)-04\Optimised Test\306(A)- Ti4AlB2N optimised\306(A)- Ti4AlB2N optimised.xrdml
Comment Configuration=Flat Sample Stage, Owner=pc, Creation date=11/22/2023 7:32:59 PM
Goniometer=Theta/Theta; Minimum step size 2Theta:0.0001; Minimum step size Omega:0.0001
Sample stage=Stage for flat samples/holders
Diffractometer system=EMPYREAN
Measurement program=C:\PANalytical\Data Collector\Programs\2B Ti3AlB2 Optimized.xrdmp, Created identifier={1511DE26-B7FE-4D9E-AD02-FB0BF5A869E9}, Created by=pc, Id=WL:DESKTOP-BC0RBBD:pc, Creation date=1/18/2024 6:31:02 PM, Modified identifier={22A65866-113A-4DB0-8C53-0AFA2CABFF67}, Modified by=pc, Id=WL:DESKTOP-BC0RBBD:pc, Modification date=2/7/2024 5:10:15 PM
PHD Lower Level = 4.02 (keV), PHD Upper Level = 16.10 (keV)

Measurement Start Date/Time	2/7/2024 5:11:00 PM
Operator	pc
Raw Data Origin	XRD measurement (*.XRDML)
Scan Axis	Gonio
Start Position [°2θ]	20.0113
End Position [°2θ]	79.9543
Step Size [°2θ]	0.0530
Scan Step Time [s]	14.2800
Scan Type	Continuous
PSD Mode	Scanning
PSD Length [°2θ]	3.35
Offset [°2θ]	0.0000
Divergence Slit Type	Fixed
Divergence Slit Size [°]	0.7197
Specimen Length [mm]	10.00
Measurement Temperature [°C]	25.00
Anode Material	Cu
Intended Wavelength Type	K-α1
K-α1 [Å]	1.54060
K-α2 [Å]	1.54443
K-β1 [Å]	1.39225
K-β2 [Å]	1.38113
K-β3 [Å]	1.39261
K-A2 / K-A1 Ratio	0.50000
K-Alpha2 Line Shift	0.00000
K Absorption Edge	1.37868
Generator Settings	40 mA, 45 kV

Diffractometer Type	0000000011286122
Diffractometer Number	0
Goniometer Radius [mm]	240.00
Dist. Focus-Diverg. Slit [mm]	60.50
Incident Beam Monochromator	No
Spinning	No

Main Graphics, Analyze View: (Bookmark 2)

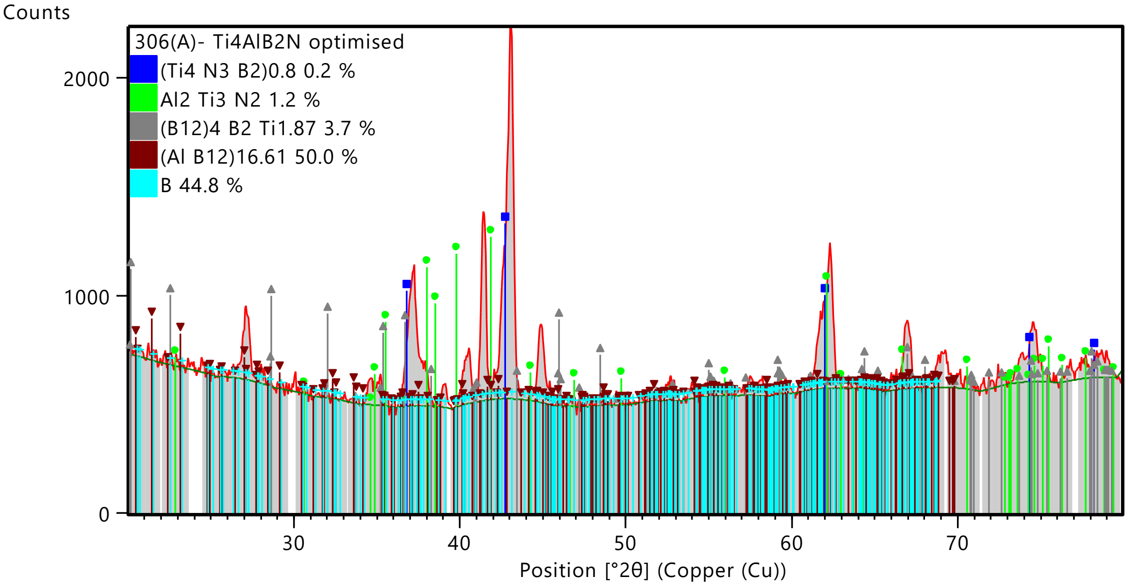

Peak List: (Bookmark 3)

Pattern List: (Bookmark 4)

Visible	Ref.Code	Score	Compound Name	Displ.[°2θ]	Scale Fac.	Chem. Formula
*	96-151-1346	62	(Ti4 N3 B2)0.8	0.000	0.359	N2.40 B1.60 Ti3.20
*	96-154-0933	Unmatched Strong	Al2 Ti3 N2	0.000	0.637	N4.00 Ti6.00 Al4.00
*	96-210-5427	3	(B12)4 B2 Ti1.87	0.000	0.528	B50.00 Ti1.87
*	96-152-4661	5	(Al B12)16.61	0.000	2.299	B176.00 Al13.36
*	96-151-1439	No Matching Lines	1511438	0.000	0.000	B189.88

Document History: (Bookmark 5)

ESD calculated from counts:
- Modification time = "2/13/2024 11:20:35 PM"
- Modification editor = "pc"

Insert Measurement:
- File name = "306(A)- Ti4AlB2N optimised.xrdml"
- Modification time = "2/13/2024 11:20:35 PM"
- Modification editor = "pc"

Default properties:
- Measurement step axis = "None"
- Internal wavelengths used from anode material: Copper (Cu)
- Original K-Alpha1 wavelength = "1.54060"
- Used K-Alpha1 wavelength = "1.54060"
- Original K-Alpha2 wavelength = "1.54443"
- Used K-Alpha2 wavelength = "1.54443"
- Original K-Beta wavelength = "1.39225"
- Used K-Beta wavelength = "1.39225"
- Irradiated length = "10.00000"
- Spinner used = "No"
- KBeta filter material = "Ni"
- KBeta filter thickness = "0.02000"
- Receiving slit size = "0.10000"
- Step axis value = "0.00000"
- Offset = "0.00000"
- Sample length = "10.00000"
- Modification time = "2/13/2024 11:20:35 PM"
- Modification editor = "pc"

Interpolate Step Size:
- Initial Scan Range = 7.02626 - 89.95710
- Initial Step Size = 0.05252
- Derived Step Size = 0.05300
- Use Derived Step Size = "Yes"
- Parameterset name = "Default"
- PANalytical factory default
- Modification time = "2/13/2024 11:20:36 PM"
- Modification editor = "pc"

Clip Range:
- Old/New start = "7.0263/20.0000"
- Old/New end = "89.9183/80.0000"
- Modification time = "2/13/2024 11:21:01 PM"
- Modification editor = "pc"

Determine Background:
- Add to net scan = "Nothing"

- User defined intensity = "-5"
- Correction method = "Automatic"
- Bending factor = "1"
- Minimum significance = "0.7"
- Minimum tip width = "0"
- Maximum tip width = "1"
- Peak base width = "2"
- Use smoothed input data = "Yes"
- Granularity = "10"
- Search window = "5"
- Spline type = "Linear"
- Parameterset name = "Untitled"
- Parameterset modification time = "2/9/2024 8:08:11 PM"
- Parameterset modification editor = "pc"
- Modification time = "2/13/2024 11:21:16 PM"
- Modification editor = "pc"

Search & Match:
- Data Source = Profile
- Auto residue = "Yes"
- Data source = "Profile and peak list"
- Demote unmatched strong = "Yes"
- Multi phase = "Yes"
- Restriction set = "Untitled"
- Restriction = "Restriction set"
- Subset name = ""
- Match intensity = "Yes"
- Two theta shift = "0"
- Identify = "No"
- Max. no. of accepted patterns = "5"
- Minimum score = "50"
- Min. new lines / total lines = "60"
- Search depth = "10"
- Minimum new lines = "5"
- Minimum scale factor = "0.1"
- Intensity threshold = "0"
- Use line clustering = "Yes"
- Line cluster range = "1.5"
- Search sensitivity = "1.8"
- Use adaptive smoothing = "Yes"
- Smoothing range = "1.5"
- Threshold factor = "3"
- Match Threshold = "0"
- N * Esds = "-1"
- Raw Weight = "-1"
- Peak Shape = "-1"
- Accepted Shape = "-1"
- Peak Power = "-1"
- New Peak Power = "-1"
- Intensity Power = "-1"
- N Peaks Power = "-1"

- Parameterset name = "Untitled"
- Parameterset modification time = "2/13/2024 11:21:52 PM"
- Parameterset modification editor = "pc"
- Modification time = "2/13/2024 11:21:55 PM"
- Modification editor = "pc"

Convert Ref. Pattern to Phase:
- Modification time = "2/13/2024 11:24:31 PM"
- Modification editor = "pc"

Edit 1511438 Title:
- Old Value = "1511438"
- Modification time = "2/13/2024 11:25:08 PM"
- Modification editor = "pc"

Edit Solver Tolerance:
- Old Value = "0.001"
- Modification time = "2/13/2024 11:25:28 PM"
- Modification editor = "pc"

Edit (Ti4 N3 B2)0.8 Asymmetry Type:
- Old Value = "No Asymmetry Function"
- Modification time = "2/13/2024 11:25:44 PM"
- Modification editor = "pc"

Edit Al2 Ti3 N2 Asymmetry Type:
- Old Value = "No Asymmetry Function"
- Modification time = "2/13/2024 11:25:53 PM"
- Modification editor = "pc"

Edit (B12)4 B2 Ti1.87 Asymmetry Type:
- Old Value = "No Asymmetry Function"
- Modification time = "2/13/2024 11:26:04 PM"
- Modification editor = "pc"

Edit (Al B12)16.61 Asymmetry Type:
- Old Value = "No Asymmetry Function"
- Modification time = "2/13/2024 11:26:08 PM"
- Modification editor = "pc"

Edit B Asymmetry Type:
- Old Value = "No Asymmetry Function"
- Modification time = "2/13/2024 11:26:13 PM"
- Modification editor = "pc"

XRD Measurement Information: (Bookmark 6)

More items… (Bookmark 7)

More items… (Bookmark 8)

More items… (Bookmark 9)

More items… (Bookmark 10)

More items… (Bookmark 11)

More items… (Bookmark 12)

More items… (Bookmark 13)

More items… (Bookmark 14)

More items… (Bookmark 15)
