# Supplementary material for: Exploring semiconductor potential: novel boron-based Ti3AlC2 and Ti4AlN3 MAX phase composites with tunable band gaps
Source: Nanoscale Adv. 2024 Nov 12;7(1):288–309. doi: 10.1039/d4na00738g (PMC11603385; doi:10.1039/d4na00738g)
Supplement: NA-007-D4NA00738G-s007 [file NA-007-D4NA00738G-s007.pdf]

Supplementary Information (SI) for Nanoscale Advances.
This journal is © The Royal Society of Chemistry 2024

This is the simple example template containing only headers for each report item and the bookmarks. The invisible bookmarks are indicated by text between brackets.
Modify it according to your own needs and standards.

Measurement Conditions: (Bookmark 1)

Dataset Name 206(A) Ti4AlB2N Optimized_2
File name E:\XRD data files\New Analysis-Dr. Moynul-BAUET\1st slot-XRD (1325 C)-04\206(A)- Ti4AlB2N-1325C\206(A) Ti4AlB2N Optimized_2.xrdml
Comment Configuration=Flat Sample Stage, Owner=pc, Creation date=11/22/2023 7:32:59 PM
Goniometer=Theta/Theta; Minimum step size 2Theta:0.0001; Minimum step size Omega:0.0001
Sample stage=Stage for flat samples/holders
Diffractometer system=EMPYREAN
Measurement program=C:\PANalytical\Data Collector\Programs\2B Ti3AlB2 Optimized.xrdmp, Created identifier={1511DE26-B7FE-4D9E-AD02-FB0BF5A869E9}, Created by=pc, Id=WL:DESKTOP-BC0RBBD:pc, Creation date=1/18/2024 6:31:02 PM
PHD Lower Level = 7.89 (keV), PHD Upper Level = 11.27 (keV)

Measurement Start Date/Time	1/20/2024 4:47:07 PM
Operator	pc
Raw Data Origin	XRD measurement (*.XRDML)
Scan Axis	Gonio
Start Position [°2θ]	10.0096
End Position [°2θ]	89.9986
Step Size [°2θ]	0.0130
Scan Step Time [s]	18.8700
Scan Type	Continuous
PSD Mode	Scanning
PSD Length [°2θ]	3.35
Offset [°2θ]	0.0000
Divergence Slit Type	Fixed
Divergence Slit Size [°]	0.7197
Specimen Length [mm]	10.00
Measurement Temperature [°C]	25.00
Anode Material	Cu
Intended Wavelength Type	K-α1
K-α1 [Å]	1.54060
K-α2 [Å]	1.54443
K-β1 [Å]	1.39225
K-β2 [Å]	1.38113
K-β3 [Å]	1.39261
K-A2 / K-A1 Ratio	0.50000
K-Alpha2 Line Shift	0.00000
K Absorption Edge	1.37868
Generator Settings	40 mA, 45 kV
Diffractometer Type	0000000011286122
Diffractometer Number	0
Goniometer Radius [mm]	240.00

Dist. Focus-Diverg. Slit [mm]	60.50
Incident Beam Monochromator	No
Spinning	No

Main Graphics, Analyze View: (Bookmark 2)

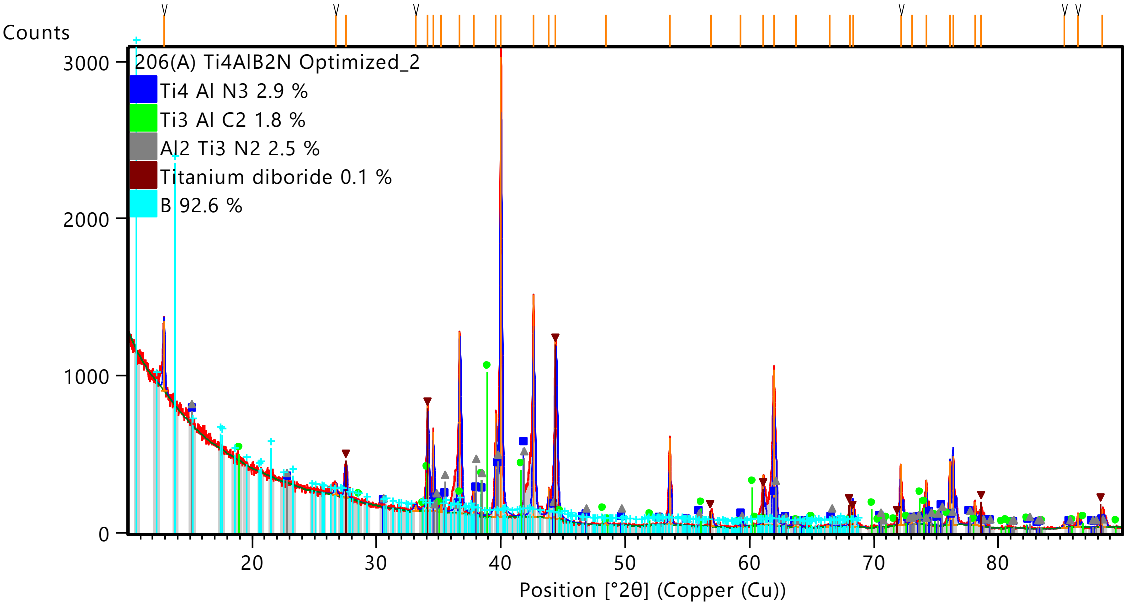

Peak List: (Bookmark 3)

Pos. [°2θ]	Height [cts]	FWHM Left [°2θ]	d-spacing [Å]	Rel. Int. [%]
12.9102	446.68	0.1279	6.85171	15.27
26.7162	74.26	0.2047	3.33410	2.54
27.5222	215.42	0.1791	3.23826	7.36
33.1225	40.78	0.3070	2.70242	1.39
34.1185	684.17	0.1535	2.62578	23.39
34.5747	501.91	0.1279	2.59217	17.16
35.2079	138.15	0.1535	2.54698	4.72
36.6929	1156.48	0.2047	2.44724	39.53
37.8314	171.91	0.1791	2.37617	5.88
39.5894	653.62	0.1279	2.27461	22.34
39.9987	2925.62	0.1535	2.25228	100.00
42.6296	1408.83	0.1535	2.11917	48.15
43.8405	186.94	0.1023	2.06340	6.39
44.4061	1157.44	0.2303	2.03842	39.56
48.4298	17.73	0.3070	1.87805	0.61
53.5799	551.21	0.1023	1.70904	18.84
56.9159	93.57	0.1791	1.61653	3.20
59.2970	64.24	0.2047	1.55717	2.20
61.1169	323.89	0.1279	1.51509	11.07
61.9515	993.11	0.1791	1.49667	33.95
63.7580	33.05	0.5117	1.45855	1.13

66.4306	13.56	0.5117	1.40620	0.46
68.0575	157.82	0.1248	1.37650	5.39
68.3099	156.90	0.1535	1.37202	5.36
72.1583	390.98	0.1279	1.30802	13.36
73.0482	37.61	0.3070	1.29427	1.29
74.2250	284.62	0.1535	1.27664	9.73
76.1255	405.71	0.1248	1.24942	13.87
76.3931	434.97	0.1279	1.24571	14.87
78.1287	149.92	0.1279	1.22232	5.12
78.6055	139.12	0.1279	1.21610	4.76
85.3293	33.48	0.2558	1.13663	1.14
86.4260	86.88	0.1279	1.12500	2.97
88.3698	114.19	0.2047	1.10520	3.90

Pattern List: (Bookmark 4)

Visible	Ref.Code	Score	Compound Name	Displ.[°2θ]	Scale Fac.	Chem. Formula
*	96-152-6339	10	Ti4 Al N3	0.000	0.141	Al2.00 N6.00 Ti8.00
*	96-722-1325	4	Ti3 Al C2	0.000	0.297	Ti6.00 C4.00 Al2.00
*	96-154-0933	7	Al2 Ti3 N2	0.000	0.229	N4.00 Ti6.00 Al4.00
*	96-200-2801	66	Titanium diboride	0.000	0.359	Ti1.00 B2.00
*	96-151-1439	0	1511438	0.000	8.651	B189.88

Document History: (Bookmark 5)

ESD calculated from counts:
- Modification time = "2/14/2024 12:49:46 AM"
- Modification editor = "pc"

Insert Measurement:
- File name = "206(A) Ti4AlB2N Optimized_2.xrdml"
- Modification time = "2/14/2024 12:49:46 AM"
- Modification editor = "pc"

Default properties:
- Measurement step axis = "None"
- Internal wavelengths used from anode material: Copper (Cu)
- Original K-Alpha1 wavelength = "1.54060"
- Used K-Alpha1 wavelength = "1.54060"
- Original K-Alpha2 wavelength = "1.54443"
- Used K-Alpha2 wavelength = "1.54443"
- Original K-Beta wavelength = "1.39225"
- Used K-Beta wavelength = "1.39225"
- Irradiated length = "10.00000"

- Spinner used = "No"
- KBeta filter material = "Ni"
- KBeta filter thickness = "0.02000"
- Receiving slit size = "0.10000"
- Step axis value = "0.00000"
- Offset = "0.00000"
- Sample length = "10.00000"
- Modification time = "2/14/2024 12:49:46 AM"
- Modification editor = "pc"

Interpolate Step Size:
- Initial Scan Range = 7.00657 - 90.00310
- Initial Step Size = 0.01313
- Derived Step Size = 0.01300
- Use Derived Step Size = "Yes"
- Parameterset name = "Default"
- PANalytical factory default
- Modification time = "2/14/2024 12:49:46 AM"
- Modification editor = "pc"

Clip Range:
- Old/New start = "7.0066/10.0000"
- Old/New end = "89.9986/89.9986"
- Modification time = "2/14/2024 12:52:39 AM"
- Modification editor = "pc"

Determine Background:
- Add to net scan = "Nothing"
- User defined intensity = "-5"
- Correction method = "Automatic"
- Bending factor = "1"
- Minimum significance = "0.7"
- Minimum tip width = "0"
- Maximum tip width = "1"
- Peak base width = "2"
- Use smoothed input data = "Yes"
- Granularity = "10"
- Search window = "5"
- Spline type = "Linear"
- Parameterset name = "Untitled"
- Parameterset modification time = "2/9/2024 8:08:11 PM"
- Parameterset modification editor = "pc"
- Modification time = "2/14/2024 12:52:48 AM"
- Modification editor = "pc"

Search Peaks:
- Minimum significance = "2"
- Minimum tip width = "0.1"
- Maximum tip width = "1"
- Peak base width = "2"
- Method = "Minimum 2nd derivative"

- Parameterset name = "Untitled"
- Parameterset modification time = "2/13/2024 3:42:23 PM"
- Parameterset modification editor = "pc"
- Modification time = "2/14/2024 12:52:58 AM"
- Modification editor = "pc"

Search & Match:
- Allow pattern shift = "No"
- Auto residue = "Yes"
- Data source = "Profile and peak list"
- Demote unmatched strong = "Yes"
- Multi phase = "Yes"
- Restriction set = "Untitled"
- Restriction = "Restriction set"
- Subset name = ""
- Match intensity = "Yes"
- Two theta shift = "0"
- Identify = "No"
- Max. no. of accepted patterns = "5"
- Minimum score = "50"
- Min. new lines / total lines = "60"
- Search depth = "10"
- Minimum new lines = "5"
- Minimum scale factor = "0.1"
- Intensity threshold = "0"
- Use line clustering = "Yes"
- Line cluster range = "1.5"
- Search sensitivity = "1.8"
- Use adaptive smoothing = "Yes"
- Smoothing range = "1.5"
- Threshold factor = "3"
- Match Threshold = "0"
- N * Esds = "-1"
- Raw Weight = "-1"
- Peak Shape = "-1"
- Accepted Shape = "-1"
- Peak Power = "-1"
- New Peak Power = "-1"
- Intensity Power = "-1"
- N Peaks Power = "-1"
- Parameterset name = "Untitled"
- Parameterset modification time = "2/14/2024 12:53:43 AM"
- Parameterset modification editor = "pc"
- Modification time = "2/14/2024 12:53:48 AM"
- Modification editor = "pc"

Convert Ref. Pattern to Phase:
- Modification time = "2/14/2024 12:59:53 AM"
- Modification editor = "pc"

Edit 1511438 Title:

- Old Value = "1511438"
- Modification time = "2/14/2024 1:00:09 AM"
- Modification editor = "pc"

Edit Solver Tolerance:
- Old Value = "0.001"
- Modification time = "2/14/2024 1:00:25 AM"
- Modification editor = "pc"

Edit Ti4 Al N3 Asymmetry Type:
- Old Value = "No Asymmetry Function"
- Modification time = "2/14/2024 1:00:34 AM"
- Modification editor = "pc"

Edit Ti3 Al C2 Asymmetry Type:
- Old Value = "No Asymmetry Function"
- Modification time = "2/14/2024 1:00:38 AM"
- Modification editor = "pc"

Edit Al2 Ti3 N2 Asymmetry Type:
- Old Value = "No Asymmetry Function"
- Modification time = "2/14/2024 1:00:43 AM"
- Modification editor = "pc"

Edit Titanium diboride Asymmetry Type:
- Old Value = "No Asymmetry Function"
- Modification time = "2/14/2024 1:00:49 AM"
- Modification editor = "pc"

Edit B Asymmetry Type:
- Old Value = "No Asymmetry Function"
- Modification time = "2/14/2024 1:00:53 AM"
- Modification editor = "pc"

XRD Measurement Information: (Bookmark 6)

More items… (Bookmark 7)

More items… (Bookmark 8)

More items… (Bookmark 9)

More items… (Bookmark 10)

More items… (Bookmark 11)

More items… (Bookmark 12)

More items… (Bookmark 13)

More items… (Bookmark 14)

More items… (Bookmark 15)
