# Supplementary material for: Exploring semiconductor potential: novel boron-based Ti3AlC2 and Ti4AlN3 MAX phase composites with tunable band gaps
Source: Nanoscale Adv. 2024 Nov 12;7(1):288–309. doi: 10.1039/d4na00738g (PMC11603385; doi:10.1039/d4na00738g)
Supplement: NA-007-D4NA00738G-s009 [file NA-007-D4NA00738G-s009.pdf]

Supplementary Information (SI) for Nanoscale Advances.
This journal is © The Royal Society of Chemistry 2024

This is the simple example template containing only headers for each report item and the bookmarks. The invisible bookmarks are indicated by text between brackets.
Modify it according to your own needs and standards.

Measurement Conditions: (Bookmark 1)

Dataset Name 209B Ti4AlB2C_13.02.2024
File name E:\XRD data files\New Analysis-Dr. Moynul-BAUET\2nd slot-XRD (1050 C)-06\209-B Ti4AlB2C-1050C\209B Ti4AlB2C_13.02.2024.xrdml
Sample Identification 209B Ti4AlB2C_13.02.2024
Comment Configuration=Flat Sample Stage, Owner=pc, Creation date=11/22/2023 7:32:59 PM
Goniometer=Theta/Theta; Minimum step size 2Theta:0.0001; Minimum step size Omega:0.0001
Sample stage=Stage for flat samples/holders
Diffractometer system=EMPYREAN
Measurement program=C:\PANalytical\Data Collector\Programs\2B Ti3AlB2 Optimized.xrdmp, Created identifier={1511DE26-B7FE-4D9E-AD02-FB0BF5A869E9}, Created by=pc, Id=WL:DESKTOP-BC0RBBD:pc, Creation date=1/18/2024 6:31:02 PM, Modified identifier={706E23D6-EF3A-4959-B077-79A506FC4E68}, Modified by=pc, Id=WL:DESKTOP-BC0RBBD:pc, Modification date=2/7/2024 9:05:13 PM
PHD Lower Level = 4.02 (keV), PHD Upper Level = 16.10 (keV)

Measurement Start Date/Time	2/13/2024 4:31:15 PM
Operator	pc
Raw Data Origin	XRD measurement (*.XRDML)
Scan Axis	Gonio
Start Position [°2θ]	15.0188
End Position [°2θ]	89.9288
Step Size [°2θ]	0.0660
Scan Step Time [s]	12.7500
Scan Type	Continuous
PSD Mode	Scanning
PSD Length [°2θ]	3.35
Offset [°2θ]	0.0000
Divergence Slit Type	Fixed
Divergence Slit Size [°]	0.7197
Specimen Length [mm]	10.00
Measurement Temperature [°C]	25.00
Anode Material	Cu
Intended Wavelength Type	K-α1
K-α1 [Å]	1.54060
K-α2 [Å]	1.54443
K-β1 [Å]	1.39225
K-β2 [Å]	1.38113
K-β3 [Å]	1.39261
K-A2 / K-A1 Ratio	0.50000
K-Alpha2 Line Shift	0.00000
K Absorption Edge	1.37868
Generator Settings	40 mA, 45 kV

Diffractometer Type	0000000011286122
Diffractometer Number	0
Goniometer Radius [mm]	240.00
Dist. Focus-Diverg. Slit [mm]	60.50
Incident Beam Monochromator	No
Spinning	No

Main Graphics, Analyze View: (Bookmark 2)

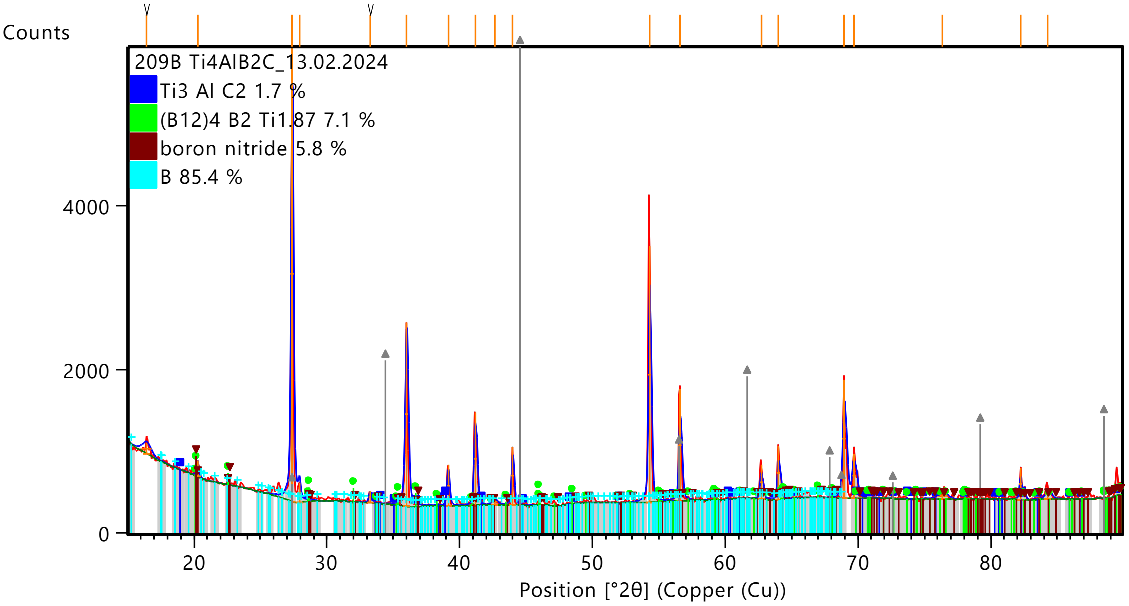

Peak List: (Bookmark 3)

Pos. [°2θ]	Height [cts]	FWHM Left [°2θ]	d-spacing [Å]	Rel. Int. [%]
16.4222	134.47	0.7793	5.39347	2.40
20.2675	195.61	0.1948	4.37803	3.49
27.3688	5611.99	0.1948	3.25607	100.00
27.9206	236.72	0.1948	3.19296	4.22
33.2668	146.48	0.1948	2.69103	2.61
36.0137	2251.88	0.2598	2.49182	40.13
39.1366	508.32	0.1948	2.29988	9.06
41.1878	1142.35	0.1948	2.18995	20.36
42.6229	79.15	0.3897	2.11948	1.41
43.9979	703.77	0.1948	2.05638	12.54
54.2830	3138.81	0.1948	1.68855	55.93
56.6058	1367.10	0.1948	1.62465	24.36
62.7092	417.73	0.1948	1.48040	7.44
64.0080	656.17	0.1948	1.45345	11.69
68.9556	1451.26	0.2598	1.36074	25.86
69.6949	542.06	0.2598	1.34811	9.66
76.3727	83.30	0.7793	1.24599	1.48
82.2629	387.48	0.1948	1.17104	6.90

84.2698	132.12	0.3897	1.14819	2.35

Pattern List: (Bookmark 4)

Visible	Ref.Code	Score	Compound Name	Displ.[°2θ]	Scale Fac.	Chem. Formula
*	96-722-1325	2	Ti3 Al C2	0.000	0.018	Ti6.00 C4.00 Al2.00
*	96-210-5427	1	(B12)4 B2 Ti1.87	0.000	0.084	B50.00 Ti1.87
*	96-153-2806	1	1532805	0.000	4.663	Al0.91 B2.00
*	96-201-6170	6	boron nitride	0.000	0.040	B48.00
*	96-151-1439	No Matching Lines	1511438	0.000	0.000	B189.88

Document History: (Bookmark 5)

ESD calculated from counts:
- Modification time = "2/14/2024 10:46:25 AM"
- Modification editor = "pc"

Insert Measurement:
- File name = "209B Ti4AlB2C_13.02.2024.xrdml"
- Modification time = "2/14/2024 10:46:25 AM"
- Modification editor = "pc"

Default properties:
- Measurement step axis = "None"
- Internal wavelengths used from anode material: Copper (Cu)
- Original K-Alpha1 wavelength = "1.54060"
- Used K-Alpha1 wavelength = "1.54060"
- Original K-Alpha2 wavelength = "1.54443"
- Used K-Alpha2 wavelength = "1.54443"
- Original K-Beta wavelength = "1.39225"
- Used K-Beta wavelength = "1.39225"
- Irradiated length = "10.00000"
- Spinner used = "No"
- KBeta filter material = "Ni"
- KBeta filter thickness = "0.02000"
- Receiving slit size = "0.10000"
- Step axis value = "0.00000"
- Offset = "0.00000"
- Sample length = "10.00000"
- Modification time = "2/14/2024 10:46:25 AM"
- Modification editor = "pc"

Interpolate Step Size:
- Initial Scan Range = 7.03283 - 89.95060
- Initial Step Size = 0.06565
- Derived Step Size = 0.06600
- Use Derived Step Size = "Yes"
- Parameterset name = "Default"
- PANalytical factory default
- Modification time = "2/14/2024 10:46:25 AM"
- Modification editor = "pc"

Clip Range:
- Old/New start = "7.0328/15.0000"
- Old/New end = "89.9288/89.9288"
- Modification time = "2/14/2024 10:47:18 AM"
- Modification editor = "pc"

Determine Background:
- Add to net scan = "Nothing"
- User defined intensity = "-5"
- Correction method = "Automatic"
- Bending factor = "1"
- Minimum significance = "0.7"
- Minimum tip width = "0"
- Maximum tip width = "1"
- Peak base width = "2"
- Use smoothed input data = "Yes"
- Granularity = "10"
- Search window = "5"
- Spline type = "Linear"
- Parameterset name = "Untitled"
- Parameterset modification time = "2/9/2024 8:08:11 PM"
- Parameterset modification editor = "pc"
- Modification time = "2/14/2024 10:47:28 AM"
- Modification editor = "pc"

Search Peaks:
- Minimum significance = "2"
- Minimum tip width = "0.1"
- Maximum tip width = "1"
- Peak base width = "2"
- Method = "Minimum 2nd derivative"
- Parameterset name = "Untitled"
- Parameterset modification time = "2/13/2024 3:42:23 PM"
- Parameterset modification editor = "pc"
- Modification time = "2/14/2024 10:47:38 AM"
- Modification editor = "pc"

Search & Match:
- Allow pattern shift = "No"
- Auto residue = "Yes"

- Data source = "Profile and peak list"
- Demote unmatched strong = "Yes"
- Multi phase = "Yes"
- Restriction set = "Untitled"
- Restriction = "Restriction set"
- Subset name = ""
- Match intensity = "Yes"
- Two theta shift = "0"
- Identify = "No"
- Max. no. of accepted patterns = "5"
- Minimum score = "50"
- Min. new lines / total lines = "60"
- Search depth = "10"
- Minimum new lines = "5"
- Minimum scale factor = "0.1"
- Intensity threshold = "0"
- Use line clustering = "Yes"
- Line cluster range = "1.5"
- Search sensitivity = "1.8"
- Use adaptive smoothing = "Yes"
- Smoothing range = "1.5"
- Threshold factor = "3"
- Match Threshold = "0"
- N * Esds = "-1"
- Raw Weight = "-1"
- Peak Shape = "-1"
- Accepted Shape = "-1"
- Peak Power = "-1"
- New Peak Power = "-1"
- Intensity Power = "-1"
- N Peaks Power = "-1"
- Parameterset name = "Untitled"
- Parameterset modification time = "2/14/2024 10:48:07 AM"
- Parameterset modification editor = "pc"
- Modification time = "2/14/2024 10:48:12 AM"
- Modification editor = "pc"

Convert Ref. Pattern to Phase:
- Modification time = "2/14/2024 10:50:44 AM"
- Modification editor = "pc"

Edit 1532805 Title:
- Old Value = "1532805"
- Modification time = "2/14/2024 10:51:10 AM"
- Modification editor = "pc"

Edit 1511438 Title:
- Old Value = "1511438"
- Modification time = "2/14/2024 10:51:17 AM"
- Modification editor = "pc"

Delete Selected Phase:
- Modification time = "2/14/2024 10:51:24 AM"
- Modification editor = "pc"

XRD Measurement Information: (Bookmark 6)

More items… (Bookmark 7)

More items… (Bookmark 8)

More items… (Bookmark 9)

More items… (Bookmark 10)

More items… (Bookmark 11)

More items… (Bookmark 12)

More items… (Bookmark 13)

More items… (Bookmark 14)

More items… (Bookmark 15)
